# A Meta-analysis of *MBL2* Polymorphisms and Tuberculosis Risk

**DOI:** 10.1038/srep35728

**Published:** 2016-11-23

**Authors:** Mohammed Y. Areeshi, Raju K. Mandal, Naseem Akhter, Sajad A. Dar, Arshad Jawed, Mohd Wahid, Harishankar Mahto, Aditya K. Panda, Mohtashim Lohani, Shafiul Haque

**Affiliations:** 1 Research and Scientific Studies Unit, College of Nursing & Allied Health Sciences, Jazan University, Jazan-45142, Saudi Arabia; 2 Department of Laboratory Medicine, Faculty of Applied Medical Sciences, Albaha University, Albaha-65431, Saudi Arabia; 3 The University College of Medical Sciences & GTB Hospital (University of Delhi), Delhi-110095, India; 4 Centre for Life Sciences, Central University of Jharkhand, Ranchi-835205, Jharkhand, India; 5 Department of Biosciences, Integral University, Lucknow-226026, Uttar Pradesh, India; 6 Department of Biosciences, Faculty of Natural Sciences, Jamia Millia Islamia (A Central University), New Delhi-110025, India

## Abstract

*MBL2* gene encodes mannose-binding lectin, is a member of innate immune system. Earlier studies revealed that *MBL2* gene variants, rs1800451, rs1800450, rs5030737, rs7096206, rs11003125 and rs7095891 are associated with impaired serum level and susceptibility to TB, but their results are inconsistent. A meta-analysis was performed by including 22 studies (7095 TB-patients and 7662 controls) and data were analyzed with respect to associations between alleles, genotypes and minor allele carriers to evaluate the potential association between *MBL2* polymorphisms and TB risk. Statistically significant results were found only for the homozygous variant genotype (CC vs. AA: p = 0.045; OR = 0.834, 95% CI = 0.699 to 0.996) of rs1800451 and showed reduced risk of TB in overall population. However, other genetic models of rs1800450, rs5030737, rs7096206, rs11003125, rs7095891 and combined rs1800450, rs1800451, rs5030737 polymorphisms of *MBL2* gene did not reveal any association with TB risk. Stratified analysis by ethnicity showed decreased risk of TB in African population for rs1800450 and rs1800451. Whereas, no association was observed between other *MBL2* polymorphisms and TB risk in all the evaluated ethnic populations. In conclusion, *MBL2* rs1800450 and rs1800451 polymorphisms play a protective role in TB infection and reinforce their critical significance as a potential genetic marker for TB resistance.

Tuberculosis (TB) is essentially caused by *Mycobacterium tuberculosis* (*M. tuberculosis*) and still a major chronic infectious disease in most parts of the world. Despite applying the various treatment strategies, which were supposed to eliminate this infectious disease from the root, but recent data revealed that TB is once again on the upsurge^1^. Worldwide, there is a heavy burden of the disease with 9.6 million new cases and 1.5 million deaths are reported in the year 2014^1^. It is assumed that nearly one-third of the world’s population is infected with *M. tuberculosis* and fairly large number of population leftover with no clinical symptoms of this infectious disease. However, nearly 5–15% of the individuals will develop the active disease as confirmed by culturable bacilli from the sputum and by other clinical symptoms^2^. Therefore, it is assumed that susceptibility and progression to active TB is partly regulated by the host genetic factors^3^, and this view is consistent with the findings of TB animal models^4^. In these infected individuals, the chances of active disease development are based on the immune system’s ability to avert the multiplication of dormant *M. tuberculosis*^5^. In this regard, the identification of host genes and genetic variations would lead to a better understanding of the pathogenesis of TB and perhaps lead to novel strategies of the treatment or prophylaxis. It is already established that innate immunity is the first line of host defense against TB. At some point during the TB infection cycle, the immunocompetent infected humans will show the presence of mycobacteria and start to generate an immune response, destroying macrophages containing bacilli. This leads to the presentation of mycobacterial antigens to the host immune system, resulting in the generation of a specific immune response against *M. tuberculosis*^6^.

The *MBL2* gene encodes for mannose-binding lectin (MBL) [also called mannose-binding protein or mannan-binding protein (MBP)], is mapped on 10q11.2-q21 chromosome, instrumental in innate immunity via the lectin pathway. MBL is a liver derived complement activating serum lectin, which binds specifically to the oligosaccharide structure present on various microorganisms comprising *M. tuberculosis*, mediates phagocytosis, and activates the mannose-binding lectin pathway of the complement^7^. Also, MBL serum concentrations are extremely variable, which are predominantly determined by several single nucleotide polymorphisms (SNPs) in both the coding and regulatory region of the *MBL2* gene^8^.

Numerous SNPs have been identified in *MBL2* gene, out of which six are acknowledged for their functional effects. Three SNPs are located at exon 1: first, rs5030737 is a C > T transition at codon 52 (CGT > TGT) that results in Arg52Cys substitution (where the variant allele is also known as ‘D’ allele), second, rs1800450 is a G > A transition (known as ‘B’ allele) at codon 54 (GGC > GAC) resulting in Gly54Asp substitution, and third, rs1800451 is a G > A transition (known as ‘C’ allele) at codon 57 (GGA > GAA) resulting in Gly57Glu substitution. These three SNPs are responsible for the disruption of the collagenous back-bone of the MBL molecule and leads to protein dysfunction^9^. Collectively, all the three SNPs (rs1800450, rs1800451, rs5030737) are designated as ‘AO’ polymorphisms and displayed alteration in the serum level of MBL. The wild type allele is designated as allele ‘A’, and the ‘O’ mutant allele indicates the presence of one or more mutant allele(s) in any of the three polymorphisms^10^. In addition to the exon 1 polymorphisms, there are three more SNPs present in the promoter region and 5′ untranslated region of the *MBL2* gene, *viz.* −550 C > G (rs11003125, also known as ‘HL’ variant, where L is the wild type allele), −221 G > C (rs7096206, ‘XY’ variant, where Y is the wild type allele) and +4 C > T (rs7095891, ‘PQ’ variant, where ‘P’ is the wild type allele), that can affect the transcription rate, and consequently the concentration of serum MBL. Up to 1000 fold variations in MBL concentration level have been noticed in different individuals possibly because of deviating actions caused due to the combination of structural genes and promoter polymorphisms^10^.

Since, all the above stated polymorphisms affect *MBL2* gene and lead to low level of the *MBL2* protein, which affects the innate immunity against the pathogen. A number of clinical (case-control) and genetic studies have been done in the past to review the impact of various *MBL2* gene polymorphism on the development of TB^11^,^12^,^13^,^14^,^15^,^16^,^17^,^18^,^19^,^20^,^21^,^22^,^23^,^24^,^25^,^26^,^27^,^28^,^29^,^30^,^31^,^32^. The results from the published studies mentioning about the role of *MBL2* gene polymorphisms and genotype on susceptibility to TB infection development are inconsistent and inconclusive. The inconsistency in the results across many of the case-control studies could possibly be due to the ethnicity of the population under consideration, sample size, and low power of the individual studies to determine the overall effect. In order to overcome this issue, nowadays a meta-analysis based statistical tool is in use to evaluate the risk factors associated with the genetic diseases, as it employs a quantitative method of pooling of the data drawn from individual studies, where small sample sizes are insufficient to deliver precise and reliable conclusions. Therefore, the present meta-analysis was performed based on literature identification until 30 March 2016 to evaluate the effect of various *MBL2* polymorphisms, rs1800451 (A > C), rs1800450 (A > B), rs5030737 (A > D), rs7096206 (Y > X), rs11003125 (H > L), rs7095891 (P > Q), and combined rs1800450, rs1800451, rs5030737 (A > O), on the risk of overall and ethnicity based effect on TB infection.

## Results

### Characteristics of the published studies

**Figure 1:**
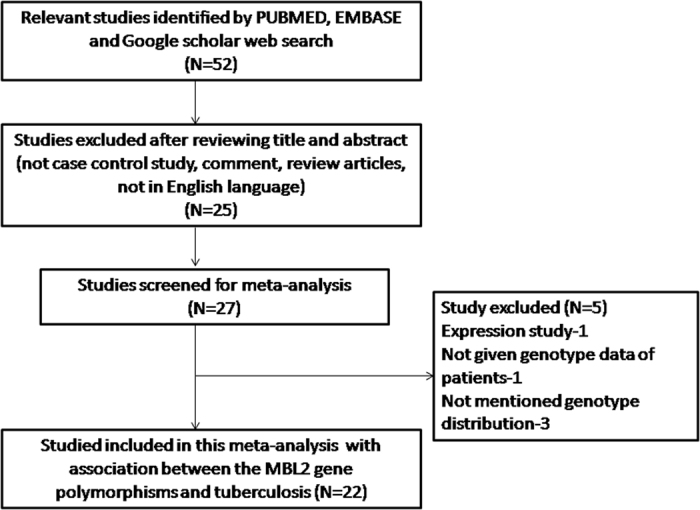
PRISMA Flow-diagram: showing identification and selection of the pertinent studies for the present meta-analysis.

The preliminary search of PubMed (Medline), EMBASE and Google Scholar web-databases resulted 52 articles using the selected key words as mentioned in the methods section, and after detailed evaluation of the titles and abstracts, and after eliminating the duplicates, 27 articles dealing with evaluation of the association of *MBL2* polymorphisms and TB risk were screened. After careful reading of the full-text of all the screened articles, 5 studies were disqualified (1 study was omitted as it reported expression analysis; 1 study was excluded due to lack of patients’ genotype data; and 3 studies were excluded because of unavailability of genotype distribution). During the study selection, all the retrieved articles were reviewed cautiously, and publications either dealing with *MBL2* variants to predict survival in TB patients or *MBL2* polymorphisms as indicator for response against therapy were disqualified. Likewise, studies pertaining to protein expression or MBL2 mRNA levels or relevant review articles were also omitted from this meta-analysis. In this meta-analysis, only case-control or cohort design studies mentioning the frequency of all the three genotypes were included. In addition to the online database search, the references given in the screened articles were also reviewed for other potential studies. Lastly, after cautious screening and following the pre-set inclusion and exclusion criteria for the selection of the studies, a total of 22 research publications^11^,^12^,^13^,^14^,^15^,^16^,^17^,^18^,^19^,^20^,^21^,^22^,^23^,^24^,^25^,^26^,^27^,^28^,^29^,^30^,^31^,^32^, showing case-control studies comprising of a total of 7095 confirmed TB patients and 7662 control subjects were included in this pooled analysis (Fig. 1 PRISMA Flow-diagram). The major characteristics of the selected studies were abstracted and have been given in Table 1. Relevant statistical data of distribution of genotypes, HWE p-values in the controls, and susceptibility towards TB have been presented in Tables 2 and 3. In order to improve the overall quality of the meta-analysis, all the 22 studies included in this meta-analysis were subjected for quality assessment following the Newcastle-Ottawa Scale (NOS) and almost all the studies (95%) scored 5 stars or more, indicating a modest to good quality (Table 4).

### Sensitivity analysis

In order to evaluate the effect of each study included in the present meta-analysis, sensitivity analysis was performed for each *MBL2* polymorphism [rs1800451 (A > C), rs1800450 (A > B), rs5030737 (A > D), Combined rs1800450, rs1800451, rs5030737 (A > O), rs7096206 (Y > X), rs11003125 (H > L), rs7095891 (P > Q)] to evaluate the influence of each individual study on the pooled OR by eliminating each single case-control study. The results of sensitivity analysis revealed that no individual study influenced the pooled ORs significantly in all the *MBL2* variants and endorsed the credibility and stability of the present meta-analysis [Supplementary Information (SI): Figures SI1–SI7].

### Quantitative synthesis

#### *MBL2* rs1800450 (A > B) polymorphism

**Figure 2:**
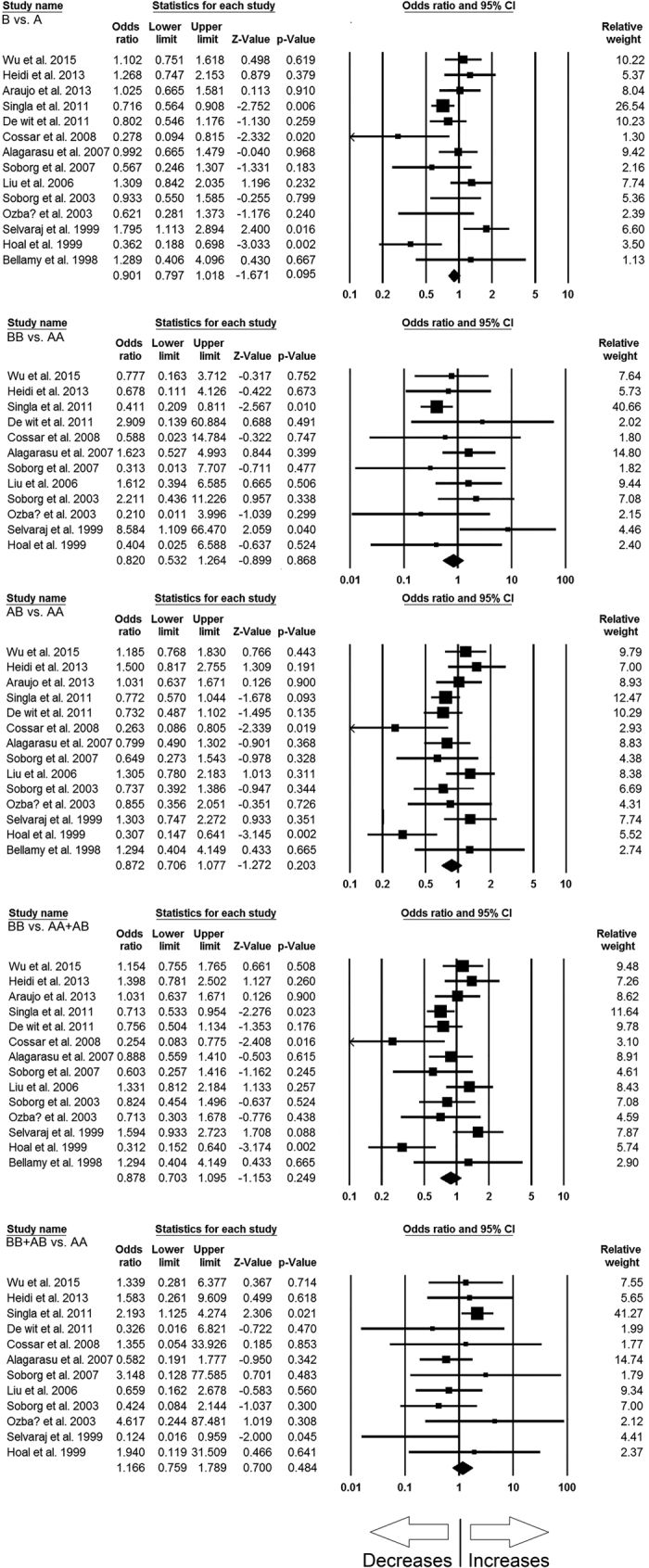
Forest plot of ORs with 95% CI of TB risk associated with the *MBL2* rs1800450 (A > B) gene polymorphism for overall population. Black square represents the value of OR and the size of the square indicates the inverse proportion relative to its variance. Horizontal line is the 95% CI of OR.

A total of 14 case-control studies comprising 2795 controls and 2993 confirmed TB cases were incorporated in this part of the analysis and provided sufficient data to calculate ORs. No publication bias was detected, whereas significant heterogeneity was observed in three genetic models (Table 5) (SI: Figure SI8). The pooled ORs revealed that *MBL2* A > B gene polymorphism is not associated with TB risk in allelic contrast (B vs. A: p = 0.095; OR = 0.901, 95% CI = 0.797 to 1.018), homozygous (BB vs. AA: p = 0.868; OR = 0.820, 95% CI = 0.532 to 1.264), heterozygous (AB vs. AA: p = 0.203; OR = 0.872, 95% CI = 0.706 to 1.077), dominant (BB + AB vs. AA: p = 0.484; OR = 1.166, 95% CI = 0.759 to 1.789), and recessive (BB vs. AA + AB: p = 0.249; OR = 0.878, 95% CI = 0.703 to 1.095) genetic models (Fig. 2).

#### *MBL2* rs1800451 (A > C) polymorphism

**Figure 3:**
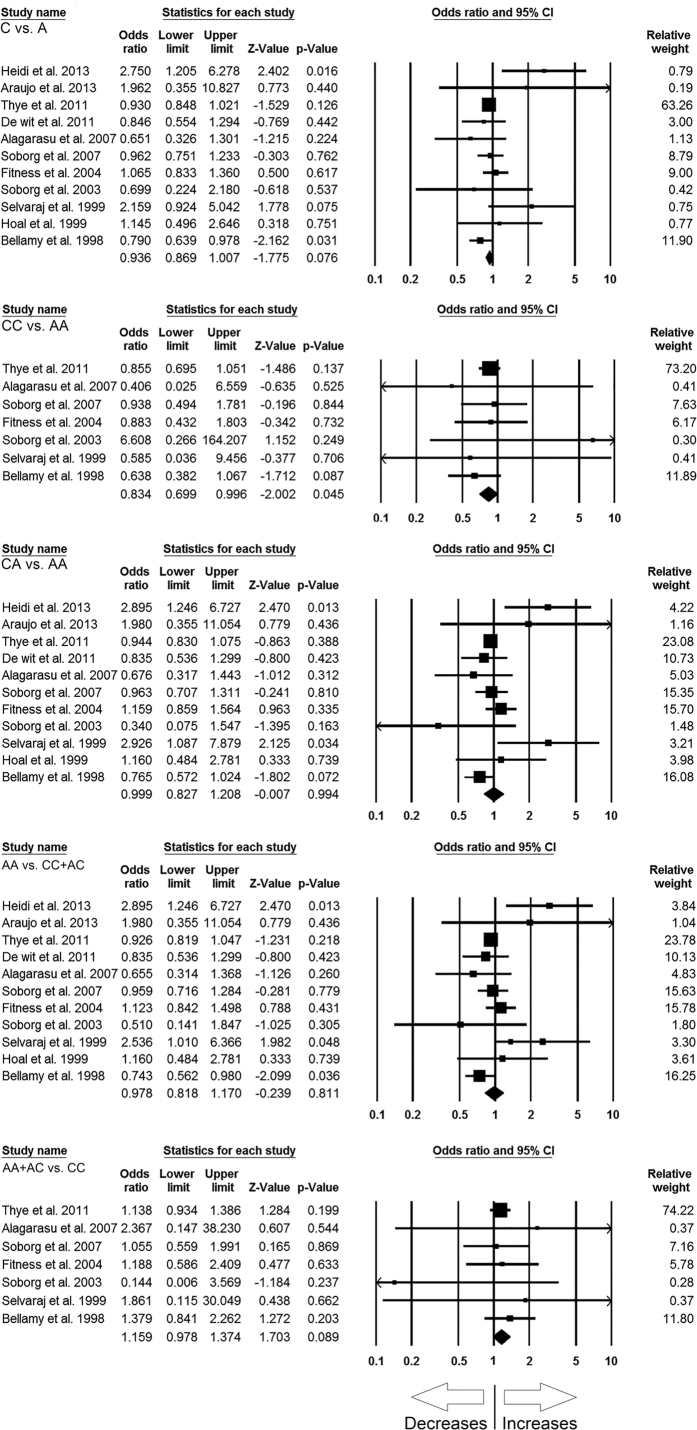
Forest plot of ORs with 95% CI of TB risk associated with the *MBL2* rs1800451 (A > C) gene polymorphism for overall population. Black square represents the value of OR and the size of the square indicates the inverse proportion relative to its variance. Horizontal line is the 95% CI of OR.

A total of 11 case-control studies containing 4535 controls and 4352 confirmed TB cases were included in this pooled study of *MBL2* A > C and TB risk, and delivered sufficient data to calculate ORs. Publication bias did not exist and heterogeneity was found in two genetic models (Table 6) (SI: Figure SI9). The pooled ORs revealed that homozygous (CC vs. AA: p = 0.045; OR = 0.834, 95% CI = 0.699 to 0.996) genetic model of *MBL2* A > C gene polymorphism is associated with reduced risk of TB (Fig. 3). Whereas, other genetic models, i.e., allelic contrast (C vs. A: p = 0.076; OR = 0.936, 95% CI = 0.869 to 1.007), heterozygous (CA vs. AA: p = 0.994; OR = 0.999, 95% CI = 0.827 to 1.208), dominant (AA + AC vs. CC: p = 0.089; OR = 1.159, 95% CI = 0.978 to 1.374), and recessive (AA vs. CC + AC: p = 0.811; OR = 0.978, 95% CI = 0.818 to 1.170) models did not show any association with TB risk (Fig. 3).

#### *MBL2* rs5030737 (A > D) polymorphism

**Figure 4:**
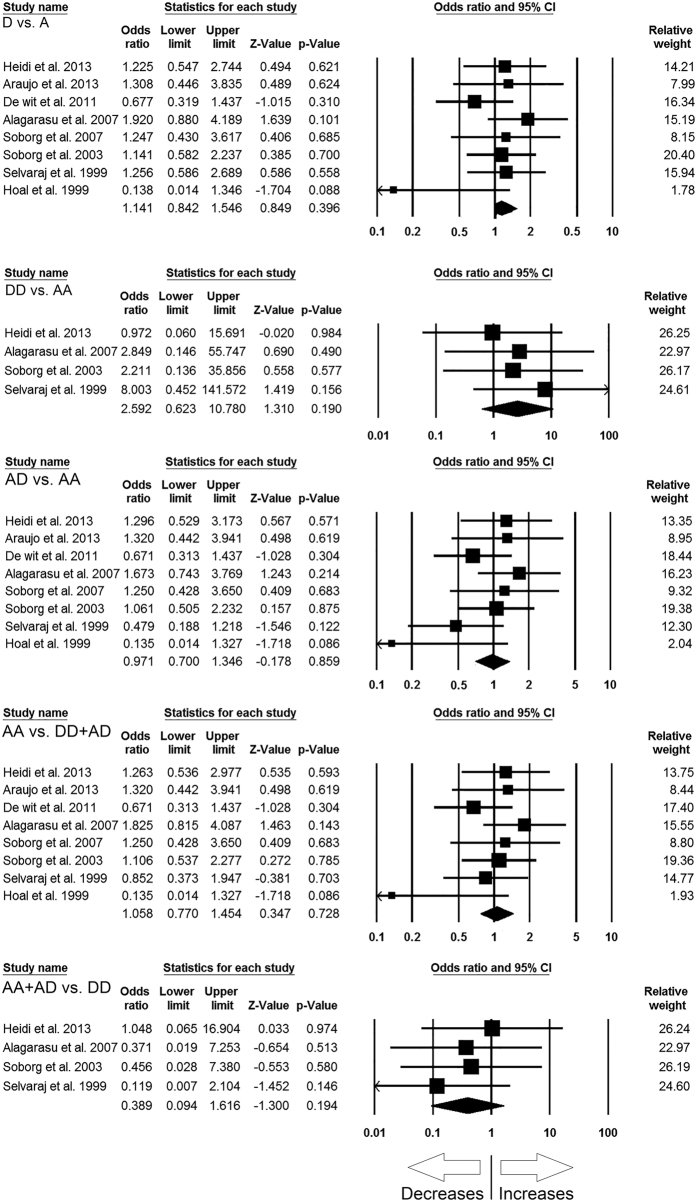
Forest plot of ORs with 95% CI of TB risk associated with the *MBL2* rs5030737 (A > D) gene polymorphism for overall population. Black square represents the value of OR and the size of the square indicates the inverse proportion relative to its variance. Horizontal line is the 95% CI of OR.

The pooled analysis for *MBL2* A > D polymorphism with TB susceptibility involved 8 case-control studies including 1191 controls and 1589 confirmed TB cases, and resulted sufficient data to calculate ORs. Publication bias and heterogeneity were not detected during the analysis (Table 7) (SI: Figure SI10). The pooled ORs revealed that *MBL2* A > D gene polymorphism was not associated with TB risk in allelic contrast (D vs. A: p = 0.396; OR = 1.141, 95% CI = 0.842 to 1.546), homozygous (DD vs. AA: p = 0.190; OR = 2.592, 95% CI = 0.623 to 10.780), heterozygous (AD vs. AA: p = 0.859; OR = 0.971, 95% CI = 0.700 to 1.346), dominant (AA + AD vs. DD: p = 0.194; OR = 0.389, 95% CI = 0.094 to 1.616), and recessive (AA vs. DD + AD: p = 0.728; OR = 1.058, 95% CI = 0.770 to 1.454) genetic models (Fig. 4).

#### *MBL2* combined rs1800450, rs1800451, rs5030737 (A > O) exon 1 polymorphism

**Figure 5:**
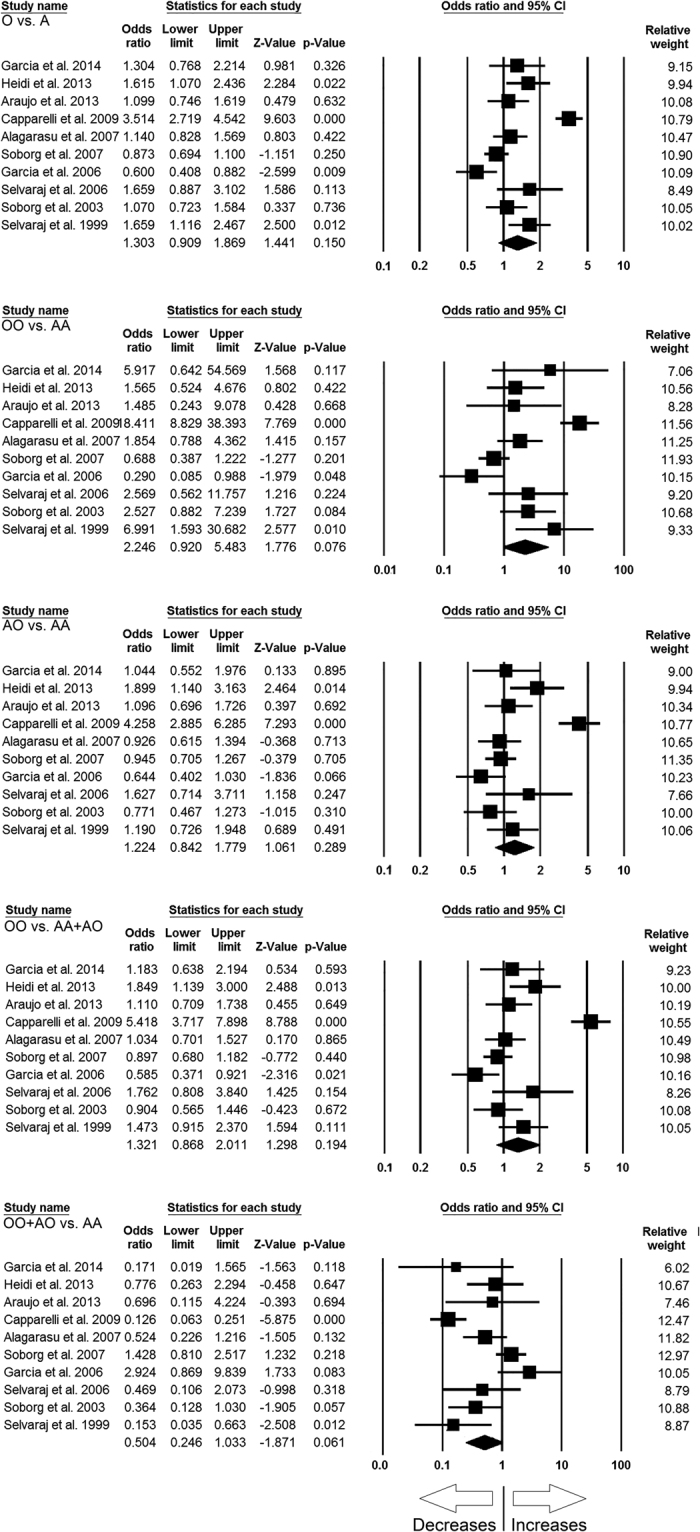
Forest plot of ORs with 95% CI of TB risk associated with the *MBL2* combined rs1800450, rs1800451, rs5030737 (A > O) exon 1 gene polymorphisms for overall population. Black square represents the value of OR and the size of the square indicates the inverse proportion relative to its variance. Horizontal line is the 95% CI of OR.

In this analysis, a total of 10 case-control studies involving 2040 control subjects and 1945 confirmed TB cases were taken into consideration and provided sufficient data to calculate ORs. No publication bias was found during the analysis, while, heterogeneity was detected in all the genetic models (Table 8) (SI: Figure SI11). The pooled ORs of this analysis revealed that combined A > O exon 1 polymorphism is not associated with TB in allelic contrast (O vs. A: p = 0.150; OR = 1.303, 95% CI = 0.909 to 1.869), homozygous (OO vs. AA: p = 0.076; OR = 2.246, 95% CI = 0.920 to 5.483), heterozygous (AO vs. AA: p = 0.289; OR = 1.224, 95% CI = 0.842 to 1.779), dominant (OO + AO vs. AA: p = 0.061; OR = 0.504, 95% CI = 0.246 to 1.033), and recessive (OO vs. AA + AO: p = 0.194; OR = 1.321, 95% CI = 0.868 to 2.011) genetic models (Fig. 5).

#### *MBL2* rs7096206 (Y > X) polymorphism

**Figure 6:**
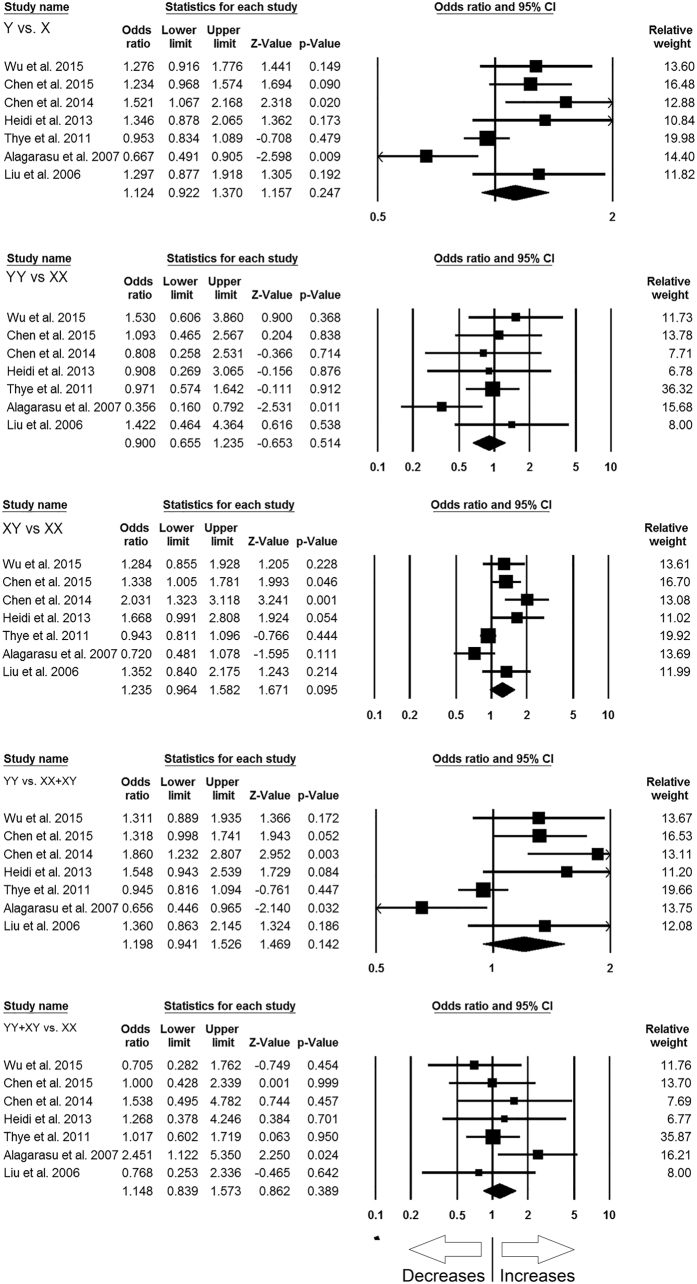
Forest plot of ORs with 95% CI of TB risk associated with the *MBL2* rs7096206 (Y > X) gene polymorphism for overall population. Black square represents the value of OR and the size of the square indicates the inverse proportion relative to its variance. Horizontal line is the 95% CI of OR.

In this analysis, a total of 7 case-control studies (3774 controls & 3379 confirmed TB cases) provided needful data to estimate ORs. Publication bias did not exist, and heterogeneity was found in three genetic models (Table 9) (SI: Figure SI12). The pooled ORs revealed that combined gene polymorphism was not associated with TB in allelic contrast (X vs. Y: p = 0.247; OR = 1.124, 95% CI = 0.922 to 1.370), homozygous (XX vs. YY: p = 0.514; OR = 0.900, 95% CI = 0.655 to 1.235), heterozygous (YX vs. XX: p = 0.095; OR = 1.235, 95% CI = 0.964 to 1.582), dominant (YY + YX vs. XX: p = 0.389; OR = 1.148, 95% CI = 0.839 to 1.573) and recessive (YY vs. XX + YX: p = 0.142; OR = 1.198, 95% CI = 0.941 to 1.526) genetic models (Fig. 6).

#### *MBL2* rs11003125 (H > L) polymorphism

**Figure 7:**
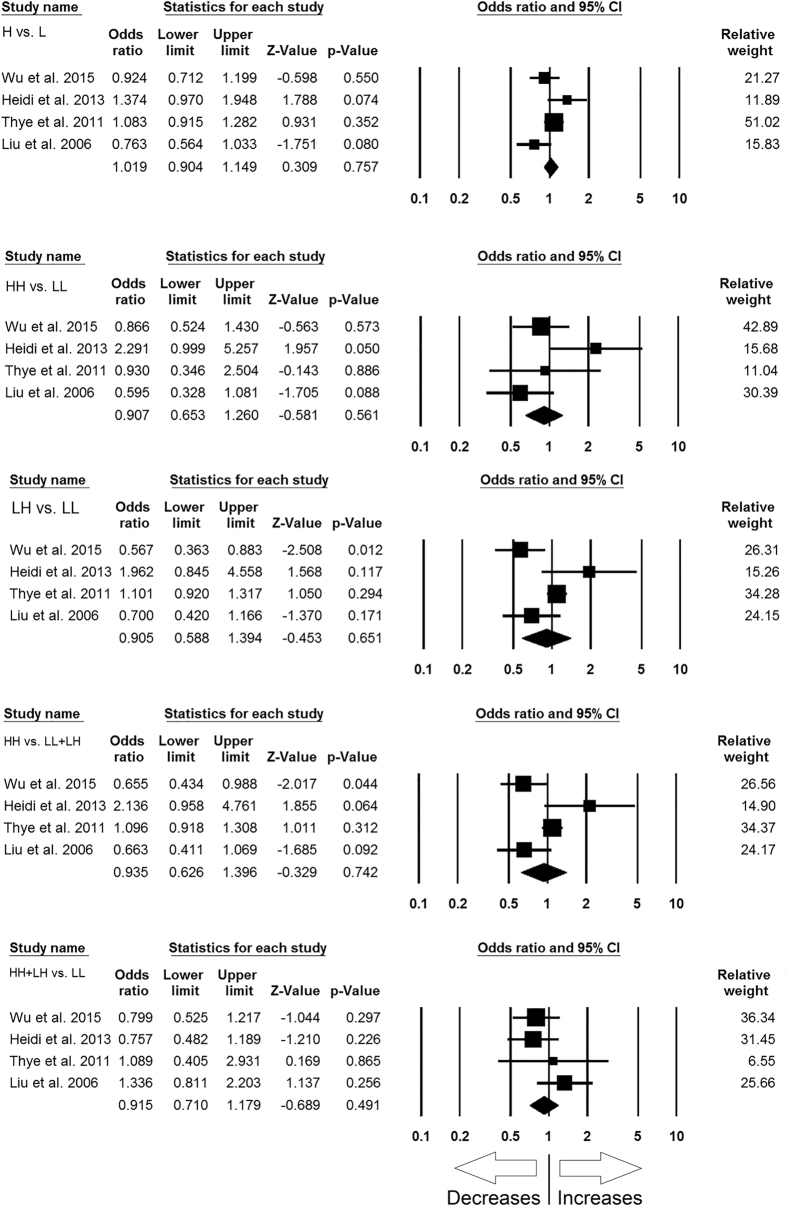
Forest plot of ORs with 95% CI of TB risk associated with the *MBL2* rs11003125 (H > L) gene polymorphism for overall population. Black square represents the value of OR and the size of the square indicates the inverse proportion relative to its variance. Horizontal line is the 95% CI of OR.

For analyzing the correlation of *MBL2* H > L gene polymorphism with TB risk, the abstracted data from 4 case-control studies involving 2989 controls and 2290 confirmed TB cases were included to calculate ORs. Publication bias did not exist, while heterogeneity was found in two genetic models (Table 10) (SI: Figure SI13). The pooled ORs demonstrated that *MBL2* H > L gene polymorphism is not associated with TB risk in allelic contrast (L vs. H: p = 0.757; OR = 1.019, 95% CI = 0.904 to 1.149), homozygous (LL vs. HH: p = 0.561; OR = 0.907, 95% CI = 0.653 to 1.260), heterozygous (HL vs. LL: p = 0.651; OR = 0.905, 95% CI = 0.588 to 1.394), dominant (HH + HL vs. LL: p = 0.491; OR = 0.915, 95% CI = 0.710 to 1.179), and recessive (HH vs. LL + HL: p = 0.742; OR = 0.935, 95% CI = 0.626 to 1.396) genetic models (Fig. 7).

#### *MBL2* rs7095891 (P > Q) polymorphism

**Figure 8:**
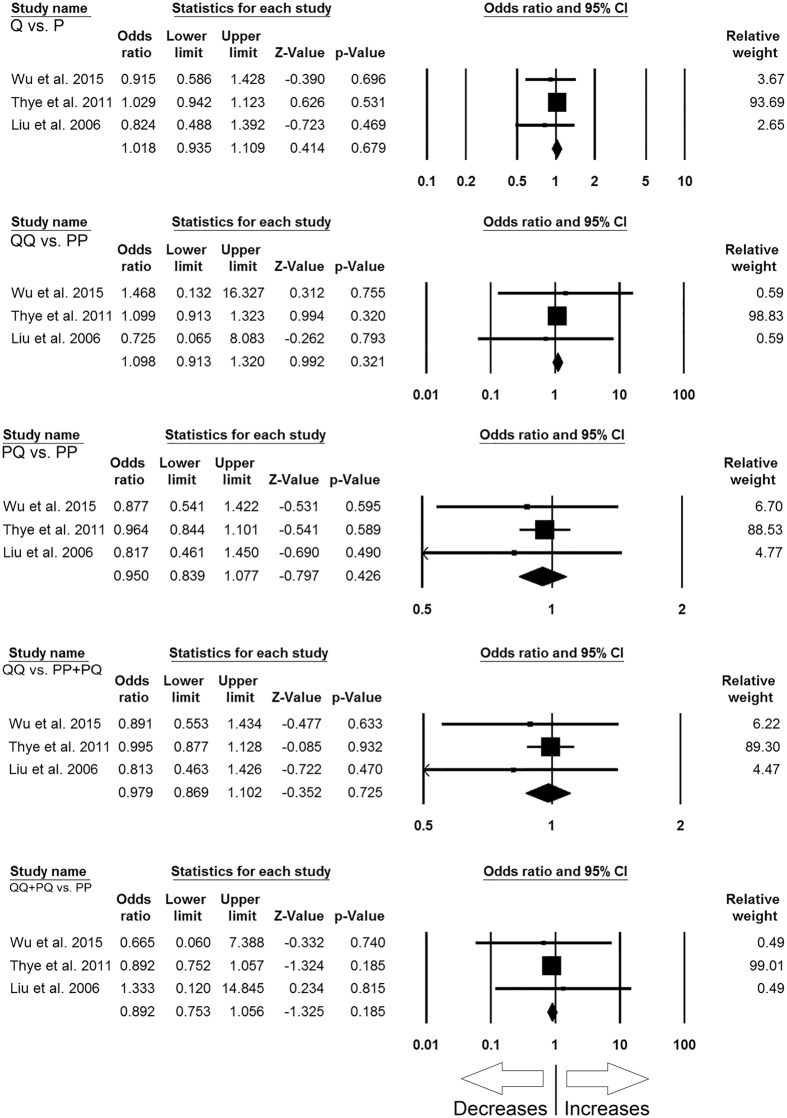
Forest plot of ORs with 95% CI of TB risk associated with the *MBL2* rs7095891 (P > Q) gene polymorphism for overall population. Black square represents the value of OR and the size of the square indicates the inverse proportion relative to its variance. Horizontal line is the 95% CI of OR.

In order to study the influence of *MBL2* P > Q gene polymorphism on TB susceptibility, this analysis was done by including 3 case-control studies involving 2895 controls and 2245 confirmed TB cases that provided the required data for the calculation of ORs. No publication bias and heterogeneity were found among all the genetic models (Table 11) (SI: Figure SI14). The pooled ORs showed that *MBL2* P > Q gene polymorphism was not associated with TB susceptibility in allelic contrast (Q vs. P: p = 0.679; OR = 1.018, 95% CI = 0.935 to 1.109), homozygous (QQ vs. PP: p = 0.321; OR = 1.098, 95% CI = 0.913 to 1.320), heterozygous (PQ vs. PP: p = 0.426; OR = 0.950, 95% CI = 0.839 to 1.077), dominant (QQ + PQ vs. PP: p = 0.185; OR = 0.892, 95% CI = 0.753 to 1.056), and recessive (QQ vs. PP + PQ: p = 0.725; OR = 0.979, 95% CI = 0.869 to 1.102) genetic models (Fig. 8).

### Subgroup stratification analysis

Subgroup stratification analysis by the ethnicity of the subjects’ (studies’) origin was performed to examine the impact of potential genetic variation on an individual’s risk of TB. The subgroup ethnicity analysis was performed when four or more case-control studies (for meaningful statistical significance) of *MBL2* polymorphisms were present in any ethnicity group. Also, only significant data have been presented here in this manuscript for all the *MBL2* polymorphisms.

As the *MBL2* combined rs1800450, rs1800451, rs5030737 (A > O) exon 1 polymorphism was reported by only three studies each from Asian, Caucasian and Mixed population, so, we failed to include those studies for the subgroup stratification analysis. Likewise, *MBL2* rs11003125 (H > L) polymorphism was reported from only two studies of Asian population and one study each of African and Mixed populations, hence we were unable to include those studies for subgroup stratification analysis. Similarly, *MBL2* rs5030737 (out of total eight studies, three each were from African and Mixed population, and two were from Asian population) and rs7095891 (out of total three studies, two were from Asian population and one was from African population) polymorphisms were not considered for the subgroup stratification analysis due to unavailability of sufficient number of studies.

#### Subgroup stratification analysis for MBL2 rs1800450

**Figure 9:**
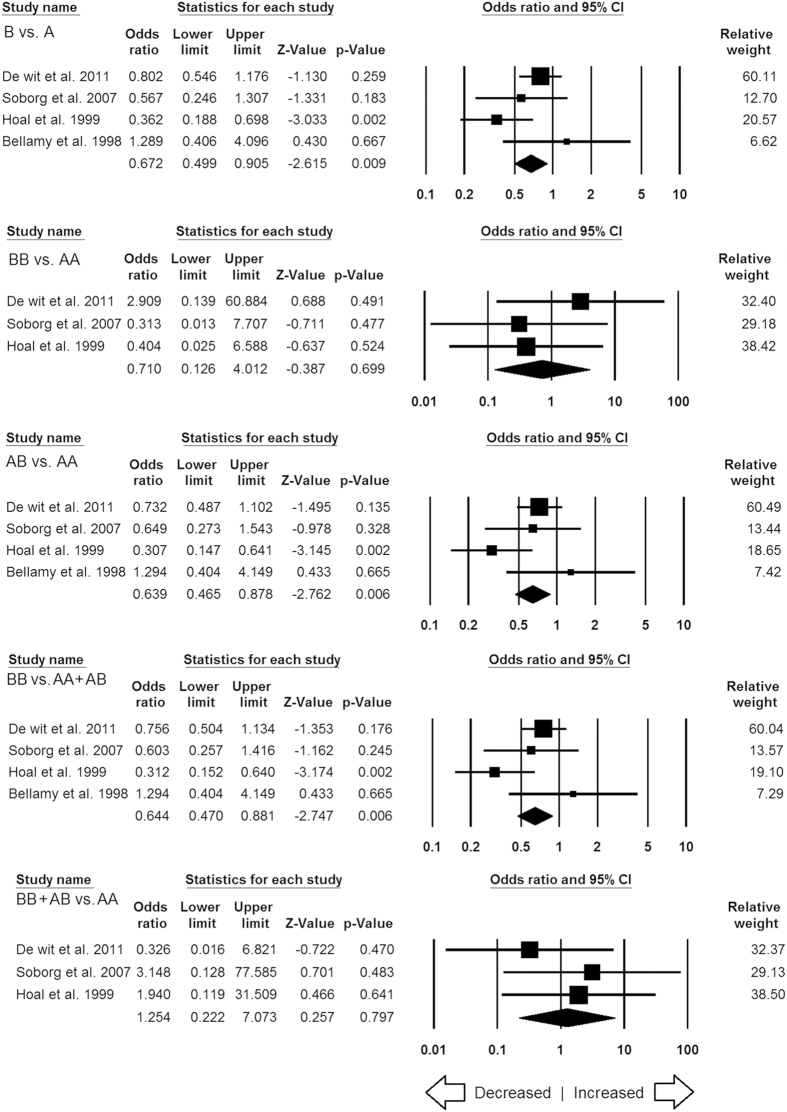
Forest plot of ORs with 95% CI of TB risk associated with the *MBL2* rs1800450 (A > B) gene polymorphism for African population. Black square represents the value of OR and the size of the square indicates the inverse proportion relative to its variance. Horizontal line is the 95% CI of OR.

**Figure 10:**
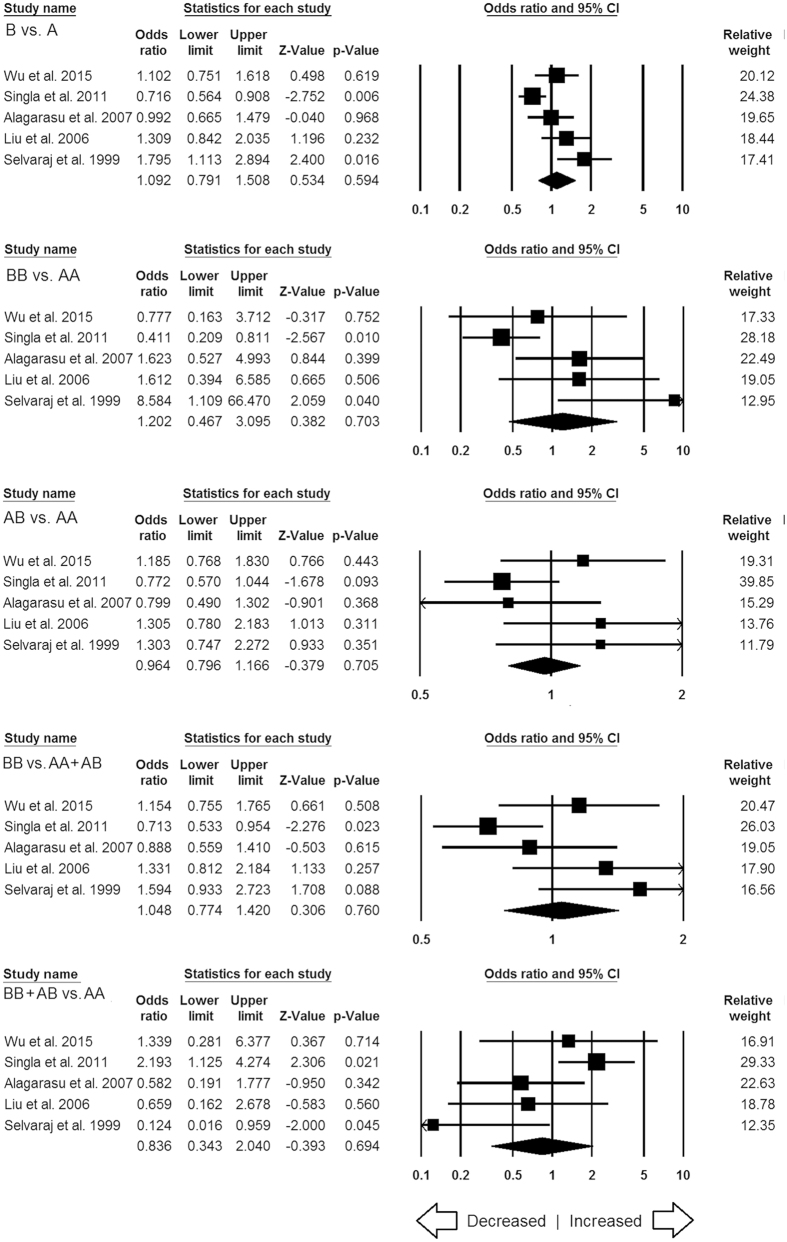
Forest plot of ORs with 95% CI of TB risk associated with the *MBL2* rs1800450 (A > B) gene polymorphism for Asian population. Black square represents the value of OR and the size of the square indicates the inverse proportion relative to its variance. Horizontal line is the 95% CI of OR.

Among the two different ethnic subgroups, Asian and African, the subgroup analysis showed no publication bias and heterogeneity among all the genetic models of African population (SI: Table ST1), whereas, publication bias and heterogeneity were noticed in some genetic models of Asian population (SI: Table ST2). The subgroup analysis demonstrated reduced risk under the allele (B vs. A: p = 0.009; OR = 0.672, 95% CI = 0.499 to 0.905), heterozygous (AB vs. AA: p = 0.006; OR = 0.639, 95% CI = 0.465 to 0.878) and dominant (BB vs. AA + AB: p = 0.006; OR = 0.644, 95% CI = 0.470 to 0.881) genetic models of African population (Fig. 9). But, no significant associations were found in case of Asian population (Fig. 10). The subgroup stratification analysis by the ethnicity was not performed for Caucasian and Mixed populations because only three studies were found from these two populations for *MBL2* rs1800450 gene variant.

#### Subgroup stratification analysis for MBL2 rs1800451

**Figure 11:**
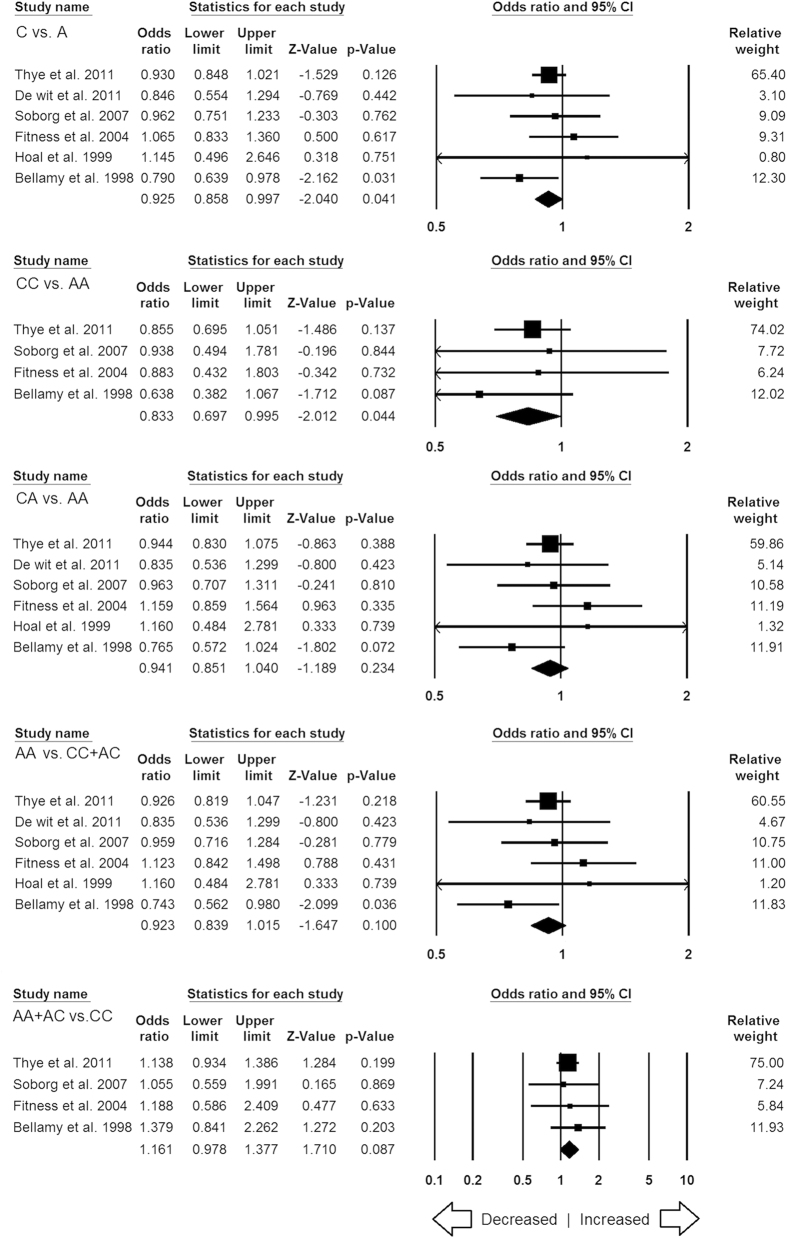
Forest plot of ORs with 95% CI of TB risk associated with the *MBL2* rs1800451 (A > C) gene polymorphism for African population. Black square represents the value of OR and the size of the square indicates the inverse proportion relative to its variance. Horizontal line is the 95% CI of OR.

In this subgroup stratification analysis, for African population, the variant C allele was associated with decreased TB risk in allele (C vs. A: p = 0.041; OR = 0.925, 95% CI = 0.858 to 0.997) and homozygous (CC vs. AA: p = 0.044; OR = 0.833, 95% CI = 0.697 to 0.995) genetic models (Fig. 11). Also, no publication bias and heterogeneity were found in all the genetic models of African population (SI: Table ST3). As only two studies were reported from Asian and three studies were originated from Mixed population, so we have not considered those studies for the ethnicity analysis.

#### Subgroup stratification analysis for MBL2 rs7096206

**Figure 12:**
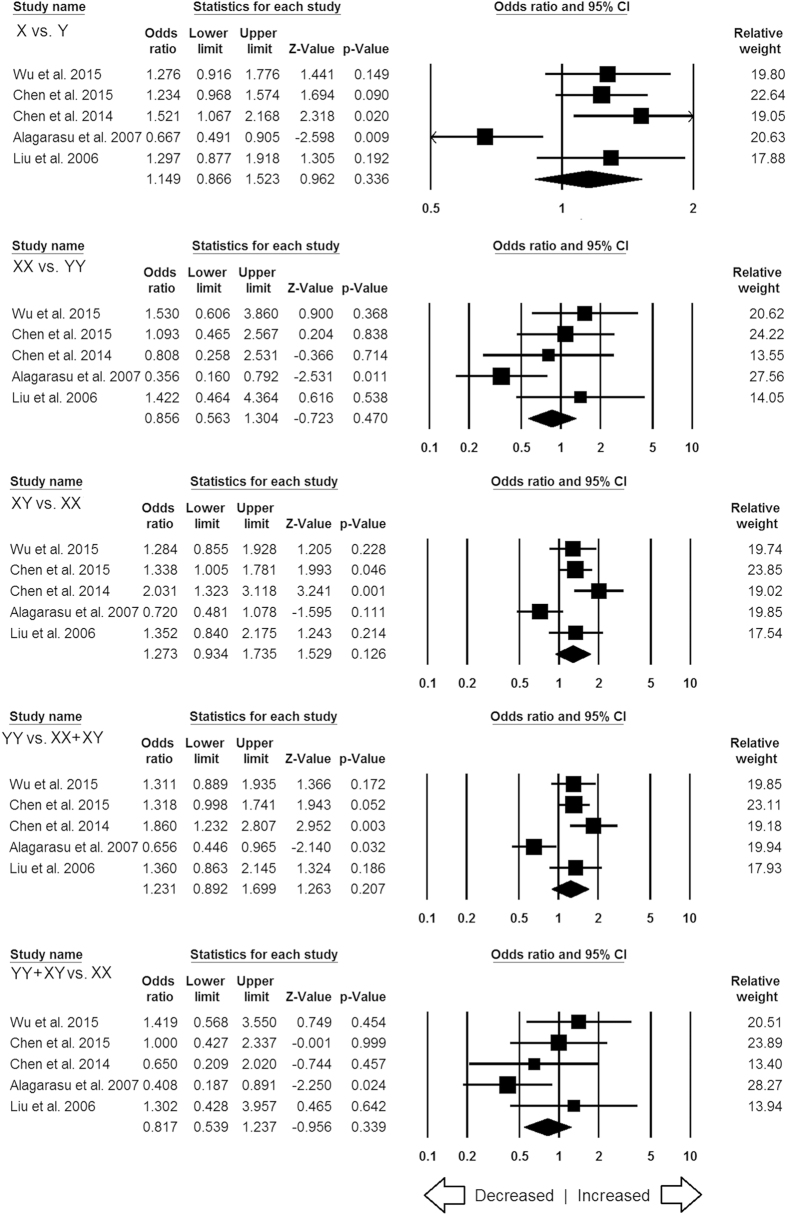
Forest plot of ORs with 95% CI of TB risk associated with the *MBL2* rs7096206 (Y > X) gene polymorphism for Asian population. Black square represents the value of OR and the size of the square indicates the inverse proportion relative to its variance. Horizontal line is the 95% CI of OR.

A total of five studies were included from Asian population for *MBL2* rs7096206 polymorphism. In this analysis, no publication bias was observed but heterogeneity was found in three genetic models of Asian population (SI: Table ST4). Overall, no significant association was found in all the five genetic models (Fig. 12). Due to lack of sufficient number of reports (only one in each ethnicity group) from African and Mixed population, studies dealing with these two populations were not considered for the subgroup stratification analysis.

## Discussion

Numerous epidemiological reports have strongly suggested the influence of genetic factors on the development of TB^33^. Likewise, earlier studies have used population based design with candidate genes of immunological pathways for their implication in TB susceptibility. Some proteins involved in innate immunity such as the MBL protein, can recognizes the mannose present on the surface of pathogens, and promotes both the opsonization and activation of the complement system. As, MBL is a calcium-dependent plasma collagenous lectin, thus plays a significant role in innate immune defense against infectious agents^34^. The gene product of MBL2 i.e., mannose-binding lectin, binds to mannose groups present on a variety of bacteria. MBP-mycobacterial complexes offer a vehicle for the dissemination by increasing the uptake through macrophages as they migrate in the blood. MBP has been shown to bind and opsonize mycobacteria, and subsequent enhanced uptake by phagocytes^35^. Further studies also reported that phosphatidylinositol mannoside of *M. tuberculosis* has been shown to bind with human MBP^36^. As, the cell wall of *M. tuberculosis* contains lipoarabinomannan (LAM), hence it has been suggested that MBP could act as a type of binding protein for mycobacteria and facilitate their entrance in the host macrophages.

Since, both the acquired and innate-immunities contribute to the killing of *M. tuberculosis*, but the precise mechanism that provide resistance against the infection has not been deciphered completely. In recent years, a number of studies have been carried out to assess exon 1, promoter, and 5′ untranslated region polymorphisms of *MBL2* gene, but their results are inconsistent. The conflicting results generated by the earlier studies may have insufficient statistical power possibly due to small sample size of the individual studies or variations that existed in different ethnicities. Therefore, in order to address the above stated limitations of the earlier case-control studies, the present meta-analysis was performed to provide a more precise statistical evidence of the association between various polymorphisms of *MBL2* gene and TB susceptibility, as the pooled ORs generated from the large sample size and sufficient statistical power from various studies have the advantage of minimizing the random errors^37^.

In this study, a total of 22 case-control studies were included for the meta-analysis which were fulfilling the pre-set eligibility criteria of the study inclusion. All the included studies clearly mentioned about the sample size, genotype, inclusion criteria of confirmed TB patients and healthy controls. We found that homozygous variant allele of rs1800451 (A > C) polymorphism was significantly associated with reduced risk of TB (Fig. 3). This indicates that individual’s carrier of homozygous genotype may be one of the factors that prevents TB patients in general population. Homozygous variant might produce low level of MBP and help the host in fighting with the infection and prevent the TB risk by showing the protective effect. These outcomes support the report of Garred *et al*.^38^, which suggests that the high frequency of MBP variant alleles in many populations might be due to higher resistance to mycobacteria^38^. However, this view requires further replications in other studies.

On the other hand, genetic models of other *MBL2* polymorphisms, i.e., rs1800450 (A > B), rs5030737 (A > D), combined polymorphisms (rs1800450, rs1800451, rs5030737: A > O), rs7096206 (Y > X), rs11003125 (H > L), rs7095891 (P > Q) were not showing any association with increased or decreased risk of TB. Earlier, Denholm *et al*.^39^, performed the meta-analysis of *MBL2* gene polymorphisms and TB susceptibility, and reported no definite and consistent association between *MBL2* genotypes and TB infection^39^. Also, the study of Denholm *et al*.^39^, has certain limitations, as many of the included studies did not report a complete *MBL2* genotype^39^. When the data were stratified by the ethnicity for the subgroup analysis, the results demonstrated that *MBL2* rs1800450 and rs1800451 polymorphisms had a protective effect of TB in African population. The subgroup analysis by the ethnicity also revealed that the strength of the association of *MBL2* polymorphisms with TB risk varied greatly across various ethnic groups. This suggests the presence of significant differences in the genetic backgrounds of different ethnic populations.

There is an emerging evidence that *M. tuberculosis* strains are genetically heterogeneous (in comparison to the previously established thought) and are correlated with specific geographical (based upon the ethnicity) areas^40^,^41^. With the passage of time, host and *M. tuberculosis* co-evolved, and individual strains are now adapted to their own specific populations^42^. It is evident from the previous studies that the risk of TB is polygenic and various genes are involved in the susceptibility of *M. tuberculosis* infection^43^. Hence, it might be possible that only *MBL2* gene polymorphisms could not be solely accountable for the predisposition of TB infection.

In the present study, significant heterogeneity was found between some of the selected studies in the test of heterogeneity. The occurrence of discord among the selected studies is possibly related to the ethnic origin of the included TB patients as ethnicity-specific genetic variations may influence the host immunity to TB infection. In addition to some concrete findings, there are some limitations associated with the present meta-analysis that need to be addressed in the future studies. First, we only included research articles published in the English language, abstracted and indexed by the specific electronic databases; it is possible that some relevant research articles appeared in other languages and indexed in some other databases may have missed. Second, the abstracted data from the included studies were not stratified by other relevant factors, such as severity of the TB infection or HIV status, and our current findings are established on unadjusted parameters. Third, we did not check for gene and environment interactions due to lack of sufficient information in the primary included studies. And, fourth, meta-analysis remains a retrospective research that is subjected to the methodological insufficiencies or selection bias of the primary included studies and may perhaps deviate or influence the reliability of the results.

Regardless of the above stated limitations, there are some advantages associated with this meta-analysis. First, this meta-analysis included more number of studies in comparison to the previously published pooled analysis, with increased statistical power and resulted statistically significant and robust conclusion. Second, publication bias did not detect. Also, the supplementary sensitivity analysis supported that the results of the present meta-analysis are reliable.

We conclude that meta-analysis is a powerful tool and gives a consensus answer using available data from different individual studies. The overall output of a meta-analysis is more precise as all the individual data is clustered during the analysis. Our result demonstrated that *MBL2* rs1800451 and rs1800451 gene polymorphisms play a protective role against the development of TB disease and might be a good candidate genetic marker for TB risk. In the near future, because of the significant public health impact of TB, definite concept, role, and mechanism of *MBL2* gene in during infection and replication of *M. tuberculosis* studies are warranted. This will assist in making complete genotype profile of the *MBL2* gene for TB susceptibility, and would significantly help in the global control and outcome of this infectious disease. Also, more comprehensive studies with group of populations considering environmental factors and HIV status are warranted to re-evaluate the associations of these *MBL2* SNPs with other gene polymorphisms in relevance with TB risk.

## Materials and Methods

### Literature search strategy

A systematic search for the pertinent studies was performed using the online databases, PubMed (Medline), EMBASE and Google Scholar covering all the research articles published with a combination of the given key words, i.e., ‘Mannose binding lectin OR *MBL2* OR MBL OR Mannose binding protein OR MBP’ gene (polymorphism OR mutation OR variant) AND tuberculosis OR TB susceptibility/risk (last updated on March 2016). We examined the potentially relevant genetic association studies by reading their titles and abstracts, and screened the most germane publication matching with the pre-set eligible criteria for a closer investigation. In addition to the web-database search, the references given in the screened research articles were also checked for other potential publications that may have been missed in the preliminary search. The search in above mentioned web-databases was only restricted to publications relating to humans.

### Inclusion and exclusion criteria

The identified studies in this meta-analysis had to meet all of the below given criteria to reduce heterogeneity and facilitate the apt interpretation of this study: (a) they must have evaluated unrelated case-control studies or cohort design between *MBL2* gene polymorphisms [rs1800451 (A > C), rs1800450 (A > C), rs5030737 (A > D), rs7096206 (Y > X), rs11003125 (H > L), rs7095891 (P > Q), and combined rs1800450, rs1800451, rs5030737 (A > O)] and TB risk, (b) should clearly designated confirmed TB patients and TB free controls, (d) have available genotype frequency in both the cases and controls to calculate odds ratio (OR) and 95% confidence interval (CI), (e) independent studies showing original data, (f) published in the English language, (g) followed statistically acceptable data collection and analysis methodology. Moreover, when the case-control study was involved in more than one research publication using the same case series, the research study that incorporated the highest number of individuals was included in this analysis. The studies were excluded based upon the following criteria: (a) duplicated or overlapping publication, (b) study design based on only TB cases, (c) without genotype distribution or allele frequency data, (d) clinical case studies without control subjects, (e) data of review, abstracts, or case reports.

### Data extraction

The methodological quality evaluation and data extraction were independently performed in duplicate copies by two independent investigators (RKM & NA) using a standard process for each retrieved article, based on the pre-set eligibility criteria of the study inclusion in the present study, and sequential exclusion of the inappropriate studies. In order to confirm the accuracy of the abstracted data by strictly following the given selection criteria a standard data-collection form was used. The following data were abstracted from each included study: publication year, the name of first author, origin country, ethnicity, source of cases and controls, number of cases and controls, type of TB [pulmonary TB (PTB) or extra-pulmonary TB (EPTB)], study type, genotype frequencies, *MBL2* polymorphisms, and reported associations. Cases of disagreement or discrepancy in any item of the abstracted data from the selected articles were resolved by conducting group discussions involving adjudicator (SAD) to accomplish a final consensus.

### Quality assessment of the selected studies

The Newcastle-Ottawa quality assessment Scale (NOS)^44^ was used independently by the two authors (RKM and NA) to evaluate the overall quality of the included studies. The NOS criteria involved three points: (1) subject selection: 0–4 points; (2) comparability of subject: 0–2 points; and (3) clinical outcome: 0–3 points. Studies scoring 5 or more stars were considered to be of moderate to high quality^44^. In order to maintain the overall quality of the meta-analysis by using NOS quality assessment, any disagreement was thoroughly discussed, and another author (adjudicator: SAD) was consulted.

### Statistical analysis

Pooled ORs and their corresponding 95% CIs were calculated to appraise the association of *MBL2* gene polymorphisms with the risk of developing TB. Chi-square-based Q-test was applied to test the heterogeneity assumption^45^, and it was considered significant at p-value < 0.05. In case of no heterogeneity, the data from single comparison was pooled by applying a fixed effects model^46^. Or else (in case of significant heterogeneity), the random-effects model was applied for the pooling of the data^47^. Also, I^2^ statistics was used to measure inter-study variability, and greater values indicated higher degree of heterogeneity^48^. Chi-square test was adopted to estimate the Hardy-Weinberg equilibrium (HWE) in the control group. Publication bias was appraised by checking the Begg’s funnel plots^49^ and Egger’s linear regression test^50^. The asymmetry of the funnel plot was measured by Egger’s regression test (a linear regression method of measuring the funnel plot asymmetry on the natural logarithm scale of the OR) and the significance of the intercept was calculated by the t-test. Statistically significant publication bias was considered at p-value < 0.05. All the software programs featuring ‘meta-analysis’ tools were compared by using the web-link: https://www.meta-analysis.com/pages/comparisons.php, and finally Biostat’s (NJ, USA) Comprehensive Meta-Analysis (CMA) Version 2 program was selected for performing all the statistical study involved in this meta-analysis.

## Additional Information

**How to cite this article**: Areeshi, M. Y. *et al*. A Meta-analysis of *MBL2* Polymorphisms and Tuberculosis Risk. *Sci. Rep.* **6**, 35728; doi: 10.1038/srep35728 (2016).

**Publisher's note:** Springer Nature remains neutral with regard to jurisdictional claims in published maps and institutional affiliations.

## Supplementary Material

Supplementary Information

## Figures and Tables

**Table 1:** Main characteristics of all studies included in the meta-analysis.

First author & Ref. No.	Study Type	Country	Ethnicity	Control	Cases	Type	*MBL2* gene variants/SNPs	Methods	Association
Wu *et al*.^11^	CC	China	Asian	453	151	PTB	P > Q, Y > X, H > L, A > B	PCR-RFLP	No
Chen *et al*.^12^	CC	China	Asian	419	503	TB	Y > X,	PCR-SSP	Yes
Chen *et al*.^13^	CC	China	Asian	216	205	TB	Y > X,	PCR-SSP	Yes
Garcia *et al*.^14^	CC	Spain	Caucasian	106	76	PTB, EPTB	A > O	PCR-SSP	No
Heidi *et al*.^15^	CC	Brazil	Mixed	148	155	PTB, EPTB	H > L, Y > X, A > O, A > D, A > B, A > C	Sequencing	A > O, A > C risk
Araujo *et al*.^16^	CC	Brazil	Mixed	159	167	PTB, EPTB	A > B, A > C, A > D, A > O	PCR	No
Singla *et al*.^17^	CC	India	Asian	392	357	PTB, EPTB	A > B	PCR-RFLP	Yes
Thye *et al*.^18^	CC	Ghana	African	2346	2230	PTB	H > L, Y > X, P > Q, A > C	Pyrosequencing	A > C
de Wit *et al*.^19^	CC	South Africa	African	318	505	TB	A>B, A > D, A > C	PCR-RFLP ARMS-PCR	No
Capparelli *et al*.^20^	CC	Italy	Caucasian	288	277	PTB	A > O	PCR	Protective
Cossar *et al*.^21^	CC	Turkey	Caucasian	44	99	PTB, EPTB	A > B	PCR-RFLP	Protective
Alagarasu *et al*.^22^	CC	India	Asian	146	366	HIV + PTB, EPTB	A > B, A > D, A > C, A > O, Y > X	PCR-SSP	Y > X, A > O
Soborg *et al*.^23^	CC	Tanzania	African	426	443	PTB	A > B, A > D, A > C, A > O	PCR-RFLP	No
Liu *et al*.^24^	CC	China	Asian	293	152	PTB	H > L, P > Q, Y > X, A > B	PCR-SSP	No
Garcia *et al*.^25^	CC	Spain	Caucasian	344	127	TB	A > O	PCR-RFLP PCR-SSP	No
Selvaraj *et al*.^26^	CC	India	Asian	58	48	PTB	A > O	PCR-SSOP	No
Fitness *et al*.^27^	CC	Malawi	African	546	322	HIV+−TB	A > C	F-PCR ARMS-PCR	Yes
Soborg *et al*.^28^	CC	Denmark	Mixed	250	109	TB	A > O, A > B, A > D, A > C	PCR-SSP	No
Ozbaş *et al*.^29^	CC	Turkey	Caucasian	100	49	PTB	A > B	PCR	No
Selvaraj *et al*.^30^	CC	India	Asian	109	202	PTB	A > B, A > C, A > D, A > O	PCR	A > C
Hoal *et al*.^31^	CC	South Africa	African	79	155	PTB, Meningeal TB	A > B, A > C, A > D	PCR-RFLP	Protective A > B
Bellamy *et al*.^32^	CC	Gambia	African	422	397	PTB	A > B, A > C	PCR	Protective A > C

Note: *MBL2 gene variants designated as: rs1800451 (A > C), rs1800450 (A > B), rs5030737 (A > D), rs7096206 (Y > X), rs11003125 (H > L), rs7095891 (P > Q), Combined rs1800450, rs1800451, rs5030737 (A > O).

**CC = Case-control study; TB = Tuberculosis; PTB = Pulmonary tuberculosis; EPTB = Extrapulmonary tuberculosis; HIV = Human immunodeficiency virus.

**Table 2:** Genotypic distribution of *MBL2* gene polymorphisms included in this meta-analysis.

First author & Ref. no.	Controls				Cases				HWE
	Genotype rs1800450			Minor allele	Genotype rs1800450			Minor allele	
	AA	AB	BB	MAF	AA	AB	BB	MAF	p-value
Wu *et al*.^11^	348	97	8	0.12	112	37	2	0.13	0.681
Heidi *et al*.^15^	124	21	3	0.09	122	31	2	0.11	0.079
Araujo *et al*.^16^	101	48	0	0.16	102	50	0	0.16	0.190
Singla *et al*.^17^	207	155	30	0.27	218	126	13	0.21	0.895
De wit *et al*.^19^	211	50	0	0.09	363	63	2	0.07	0.087
Cossar *et al*.^21^	71	27	1	0.14	40	4	0	0.04	0.366
Alagarasu *et al*.^22^	86	33	4	0.16	212	65	16	0.16	0.704
Soborg *et al*.^23^	271	13	1	0.02	289	9	0	0.01	0.063
Liu *et al*.^24^	166	42	4	0.11	103	34	4	0.14	0.487
Soborg *et al*.^28^	157	48	3	0.12	71	16	3	0.12	0.756
Ozbaş *et al*.^29^	76	20	4	0.14	40	9	0	0.09	0.090
Selvaraj *et al*.^30^	84	24	1	0.11	137	51	14	0.19	0.615
Hoal *et al*.^31^	46	21	1	0.16	114	16	1	0.06	0.414
Bellamy *et al*.^32^	183	5	0	0.01	198	7	0	0.01	0.853
	**Genotype rs1800451**				**Genotype rs1800451**				
	**AA**	**AC**	**CC**		**AA**	**AC**	**CC**		
Heidi *et al*.^15^	140	8	0	0.02	133	22	0	0.07	0.735
Araujo *et al*.^16^	101	2	0	0.00	102	4	0	0.01	0.920
Thye et al.^18^	1002	977	257	0.33	885	815	194	0.31	0.421
De wit *et al*.^19^	211	39	0	0.07	363	56	0	0.06	0.181
Alagarasu *et al*.^22^	86	12	1	0.07	212	20	1	0.04	0.439
Soborg *et al*.^23^	271	112	20	0.18	289	115	20	0.18	0.065
Fitness *et al*.^27^	362	160	24	0.19	205	105	12	0.20	0.244
Soborg *et al*.^28^	157	13	0	0.03	71	2	1	0.02	0.604
Selvaraj *et al*.^30^	103	5	1	0.03	176	25	1	0.06	0.006
Hoal *et al*.^31^	46	8	0	0.07	114	23	0	0.08	0.556
Bellamy *et al*.^32^	183	192	42	0.33	198	159	29	0.28	0.417
	**Genotype rs5030737**				**Genotype rs5030737**				
	**AA**	**AD**	**DD**		**AA**	**AD**	**DD**		
Heidi *et al*.^15^	138	9	1	0.03	142	12	1	0.04	0.067
Araujo *et al*.^16^	101	6	0	0.02	102	8	0	0.03	0.765
De wit *et al*.^19^	211	13	0	0.02	363	15	0	0.01	0.654
Alagarasu *et al*.^22^	86	8	0	0.04	212	33	3	0.07	0.666
Soborg *et al*.^23^	271	6	0	0.01	289	8	0	0.01	0.855
Soborg *et al*.^28^	157	25	1	0.07	71	12	1	0.08	0.996
Selvaraj *et al*.^30^	99	10	0	0.04	186	9	7	0.05	0.615
Hoal *et al*.^31^	46	3	0	0.03	114	1	0	0.00	0.825

MAF, Minor allele frequency, HWE, Hardy Weinberg Equilibrium.

**Table 3:** Genotypic distribution of *MBL2* gene polymorphisms included in this meta-analysis.

First author & Ref. no.	Controls				Cases				HWE
	Combined rs1800450, rs1800451, rs5030737			Minor allele	Combined rs1800450, rs1800451, rs5030737			Minor allele	
	AA	AO	OO	MAF	AA	AO	OO	MAF	p-value
Garcia *et al*.^14^	71	34	1	0.16	48	24	4	0.21	0.156
Heidi *et al*.^15^	108	34	6	0.15	92	55	8	0.22	0.128
Araujo *et al*.^16^	101	56	2	0.18	102	62	3	0.20	0.057
Capparelli *et al*.^20^	166	112	10	0.22	55	158	61	0.51	0.087
Alagarasu *et al*.^22^	86	53	7	0.22	212	121	32	0.25	0.747
Soborg *et al*.^23^	271	131	30	0.22	289	132	22	0.19	0.030
Garcia *et al*.^25^	183	134	27	0.27	70	33	3	0.18	0.721
Selvaraj *et al*.^26^	37	18	3	0.20	24	19	5	0.30	0.678
Soborg *et al*.^28^	157	86	7	0.20	71	30	8	0.21	0.235
Selvaraj *et al*.^30^	68	39	2	0.19	107	73	22	0.28	0.175
	**Genotype rs7096206**				**Genotype rs7096206**				
	**YY**	**YX**	**XX**		**YY**	**YX**	**XX**		
Wu *et al*.^11^	318	120	15	0.16	97	47	7	0.20	0.379
Chen *et al*.^12^	296	113	10	0.15	325	166	12	0.18	0.839
Chen *et al*.^13^	159	49	8	0.15	123	77	5	0.21	0.097
Heidi *et al*.^15^	110	32	6	0.14	101	49	5	0.19	0.076
Thye *et al*.^18^	1663	486	31	0.12	1437	396	26	0.12	0.502
Alagarasu *et al*.^22^	72	61	13	0.29	218	133	14	0.22	0.987
Liu *et al*.^24^	151	54	7	0.16	91	44	6	0.19	0.430
	**Genotype rs11003125**				**Genotype rs11003125**				
	**HH**	**HL**	**LL**		**HH**	**HL**	**LL**		
Wu *et al*.^11^	101	248	104	0.50	46	64	41	0.48	0.043
Heidi *et al*.^15^	19	61	68	0.66	10	63	82	0.73	0.366
Thye *et al*.^18^	1878	289	9	0.07	1570	266	7	0.07	0.550
Liu *et al*.^24^	49	105	58	0.52	44	66	31	0.45	0.911
	**Genotype rs7095891**				**Genotype rs7095891**				
	**PP**	**PQ**	**QQ**		**PP**	**PQ**	**QQ**		
Wu *et al*.^11^	364	87	2	0.10	124	26	1	0.09	0.181
Thye *et al*.^18^	825	1086	319	0.38	725	920	308	0.39	0.204
Liu *et al*.^24^	171	39	2	0.10	118	22	1	0.08	0.891

MAF, Minor allele frequency, HWE, Hardy Weinberg equilibrium.

**Table 4:** Quality assessment conducted according to the Newcastle-Ottawa Scale for all the included studies.

First author and Ref. no.	Quality indicators		
	Selection	Comparability	Exposure
Wu *et al*.^11^	***	*	**
Chen *et al*.^12^	***	*	***
Chen *et al*.^13^	***	*	***
Garcia *et al*.^14^	**	*	**
Heidi *et al*.^15^	***	*	**
Araujo *et al*.^16^	***	*	**
Singla *et al*.^17^	***	*	***
Thye *et al*.^18^	****	*	***
de Wit *et al*.^19^	***	*	**
Capparelli *et al*.^20^	***	*	**
Cossar *et al*.^21^	**	*	**
Alagarasu *et al*.^22^	***	*	*
Soborg *et al*.^23^	***	*	*
Liu *et al*.^24^	***	*	***
Garcia *et al*.^25^	****	*	**
Selvaraj *et al*.^26^	***	*	*
Fitness *et al*.^27^	****	*	**
Soborg *et al*.^28^	***	*	**
Ozbaş *et al*.^29^	*	*	**
Selvaraj *et al*.^30^	***	*	**
Hoal *et al*.^31^	****	*	**
Bellamy *et al*.^32^	**	*	**

**Table 5:** Statistics to test publication bias and heterogeneity in this meta-analysis: *MBL2* rs1800450 (A > B) polymorphism.

Comparisons	Egger’s regression analysis			Heterogeneity analysis			Model used for the meta-analysis
	Intercept	95% Confidence Interval	p-value	Q-value	P_heterogeneity_	I^2^ (%)	
B vs. A	−0.36	−2.83 to 2.10	0.75	34.32	0.002	59.78	Random
BB vs. AA	0.69	−0.86 to 2.24	0.34	14.93	0.185	26.34	Fixed
AB vs. AA	−0.65	−3.02 to 1.70	0.55	24.53	0.027	47.01	Random
BB vs. AA + AB	−0.58	−3.11 to 1.95	0.62	28.57	0.008	54.50	Random
BB + AB vs. AA	−0.65	−2.14 to 0.82	0.34	13.83	0.242	20.50	Fixed

**Table 6:** Statistics to test publication bias and heterogeneity in this meta-analysis: *MBL2* rs1800451 (A > C) polymorphism.

Comparisons	Egger’s regression analysis			Heterogeneity analysis			Model used for the meta-analysis
	Intercept	95% Confidence Interval	p-value	Q-value	P_heterogeneity_	I^2^ (%)	
C vs. A	0.64	−0.58 to 1.87	0.26	16.28	0.092	38.58	Fixed
CC vs. AA	0.06	−1.04 to 1.17	0.88	3.16	0.788	0.001	Fixed
CA vs. AA	0.58	−0.89 to 2.06	0.39	19.21	0.038	47.99	Random
AA vs. CC + AC	0.66	−0.75 to 2.09	0.31	18.61	0.045	46.28	Random
AA + AC vs. CC	−0.04	−1.04 to 0.94	0.91	2.58	0.859	0.001	Fixed

**Table 7:** Statistics to test publication bias and heterogeneity in this meta-analysis: *MBL2* rs5030737 (A > D) polymorphism.

Comparisons	Egger’s regression analysis			Heterogeneity analysis			Model used for the meta-analysis
	Intercept	95% Confidence Interval	p-value	Q-value	P_heterogeneity_	I^2^ (%)	
D vs. A	−1.77	−4.75 to 1.21	0.19	7.04	0.425	0.57	Fixed
DD vs. AA	9.67	−41.00 to 60.34	0.49	1.08	0.781	0.001	Fixed
AD vs. AA	−1.80	−5.48 to 1.87	0.27	8.67	0.27	19.27	Fixed
AA vs. DD + AD	−1.68	−4.87 to 1.50	0.24	6.94	0.43	0.001	Fixed
AA + AD vs. DD	−9.36	−62.48 to 43.76	0.52	1.15	0.76	0.001	Fixed

**Table 8:** Statistics to test publication bias and heterogeneity in the present meta-analysis: *MBL2* combined rs1800450, rs1800451, rs5030737 (A > O) polymorphism.

Comparisons	Egger’s regression analysis			Heterogeneity analysis			Model used for the meta-analysis
	Intercept	95% Confidence Interval	p-value	Q-value	P_heterogeneity_	I^2^ (%)	
O vs. A	−1.02	−9.79 to 7.75	0.79	89.83	0.001	89.98	Random
OO vs. AA	0.76	−4.59 to 6.12	0.75	62.10	0.001	85.50	Random
AO vs. AA	0.20	−7.59 to 8.00	0.95	58.38	0.001	84.58	Random
OO vs. AA + AO	0.98	−8.03 to 10.00	0.80	80.99	0.001	88.88	Random
OO + AO vs. AA	−0.82	−5.07 to 3.42	0.66	40.76	0.001	77.92	Random

**Table 9:** Statistics to test publication bias and heterogeneity in this meta-analysis: *MBL2* rs7096206 (Y > X) polymorphism.

Comparisons	Egger’s regression analysis			Heterogeneity analysis			Model used for the meta-analysis
	Intercept	95% Confidence Interval	P value	Q value	P_heterogeneity_	I^2^ (%)	
X vs. Y	2.03	−2.08 to 6.14	0.25	20.14	0.003	70.21	Random
XX vs. YY	0.36	−3.70 to 4.43	0.82	7.38	0.287	18.71	Fixed
XY vs. XX	2.41	−1.13 to 5.96	0.14	21.66	0.001	72.30	Random
YY vs. XX + XY	2.22	−1.64 to 6.10	0.19	22.22	0.001	73.00	Random
YY + XY vs. XX	0.07	−3.57 to 3.72	0.95	5.80	0.446	0.001	Fixed

**Table 10:** Statistics to test publication bias and heterogeneity in this meta-analysis: *MBL2* rs11003125 (H > L) polymorphism.

Comparisons	Egger’s regression analysis			Heterogeneity analysis			Model used for the meta-analysis
	Intercept	95% Confidence Interval	p-value	Q-value	P_heterogeneity_	I^2^ (%)	
L vs. H	−0.69	−15.13 to 13.75	0.85	7.39	0.06	59.41	Fixed
LL vs. HH	2.59	−10.78 to 15.97	0.49	6.73	0.08	55.45	Fixed
LH vs. LL	−1.00	−10.49 to 8.49	0.69	11.71	0.01	74.39	Random
HH vs. LL + LH	−0.71	10.65 to 9.22	0.78	11.37	0.01	73.61	Random
HH + LH vs. LL	1.30	−8.32 to 10.93	0.61	3.40	0.33	11.75	Fixed

**Table 11:** Statistics to test publication bias and heterogeneity in this meta-analysis: *MBL2* rs7095891 (P > Q) polymorphism.

Comparisons	Egger’s regression analysis			Heterogeneity analysis			Model used for the meta-analysis
	Intercept	95% Confidence Interval	p-value	Q-value	P_heterogeneity_	I^2^ (%)	
Q vs. P	−0.82	−3.15 to 1.50	0.13	0.89	0.63	0.001	Fixed
QQ vs. PP	−0.05	−4.02 to 3.91	0.88	0.17	0.91	0.001	Fixed
PQ vs. PP	−0.63	−2.03 to 0.75	0.10	0.41	0.81	0.001	Fixed
QQ vs. PP + PQ	−0.77	−2.70 to 1.16	0.12	0.63	0.72	0.001	Fixed
QQ + PQ vs. PP	0.04	−3.83 to 3.93	0.90	0.16	0.92	0.001	Fixed
