# Supplementary material for: A Meta-analysis of MBL2 Polymorphisms and Tuberculosis Risk
Source: Sci Rep. 2016 Nov 23;6:35728. doi: 10.1038/srep35728 (PMC5120291; doi:10.1038/srep35728)
Supplement: Supplementary Information [file srep35728-s1.pdf]

Supplementary Information

A Meta-analysis of *MBL2* Polymorphisms and Tuberculosis Risk

Authors' names in order of their authorship
Mohammed Y. Areeshi[1#], Raju K. Mandal[1#], Naseem Akhter[2], Sajad A. Dar[1,3], Arshad Jawed[1], Mohd Wahid[1,4], Harishankar Mahto[5], Aditya K. Panda[5], Mohtashim Lohani[1,6], Shafiul Haque[1,4]✉

B vs. A — Statistics with study removed

Study name	Point	Lower limit	Upper limit	Z-Value	p-Value
Wu et al. 2015	0.880	0.773	1.002	-1.932	0.053
Heidi et al. 2013	0.883	0.779	1.002	-1.927	0.054
Araujo et al. 2013	0.891	0.784	1.012	-1.776	0.076
Singla et al. 2011	0.979	0.848	1.129	-0.296	0.768
De wit et al. 2011	0.913	0.802	1.039	-1.382	0.167
Cossar et al. 2008	0.915	0.808	1.035	-1.415	0.157
Alagarasu et al. 2007	0.892	0.784	1.014	-1.743	0.081
Soborg et al. 2007	0.910	0.804	1.030	-1.491	0.136
Liu et al. 2006	0.873	0.768	0.992	-2.086	0.037
Soborg et al. 2003	0.899	0.792	1.020	-1.657	0.098
Ozba? et al. 2003	0.909	0.803	1.020	-1.507	0.132
Selvaraj et al. 1999	0.858	0.756	0.974	-2.367	0.018
Hoal et al. 1999	0.931	0.822	1.055	-1.123	0.261
Bellamy et al. 1998	0.897	0.793	1.015	-1.726	0.084
	0.901	0.797	1.018	-1.671	0.095

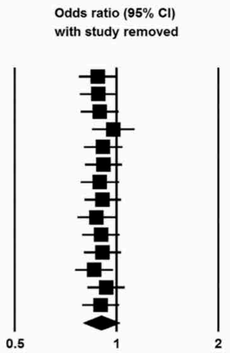

BB vs. AA — Statistics with study removed

Study name	Point	Lower limit	Upper limit	Z-Value	p-Value
Wu et al. 2015	0.824	0.525	1.292	-0.845	0.398
Heidi et al. 2013	0.830	0.531	1.295	-0.822	0.411
Singla et al. 2011	1.316	0.750	2.306	0.958	0.338
De wit et al. 2011	0.799	0.516	1.237	-1.007	0.314
Cossar et al. 2008	0.825	0.533	1.276	-0.864	0.388
Alagarasu et al. 2007	0.728	0.456	1.164	-1.326	0.185
Soborg et al. 2007	0.835	0.540	1.292	-0.811	0.417
Liu et al. 2006	0.764	0.485	1.204	-1.159	0.246
Soborg et al. 2003	0.760	0.486	1.191	-1.197	0.231
Ozba? et al. 2003	0.845	0.546	1.308	-0.755	0.450
Selvaraj et al. 1999	0.735	0.472	1.144	-1.365	0.172
Hoal et al. 1999	0.834	0.539	1.293	-0.810	0.418
	0.820	0.532	1.264	-0.899	0.368

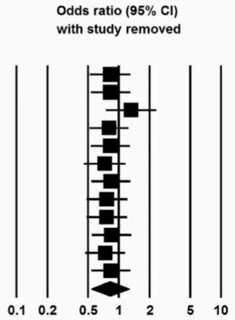

AB vs. AA — Statistics with study removed

Study name	Point	Lower limit	Upper limit	Z-Value	p-Value
Wu et al. 2015	0.843	0.674	1.055	-1.493	0.136
Heidi et al. 2013	0.839	0.679	1.037	-1.623	0.105
Araujo et al. 2013	0.856	0.680	1.077	-1.330	0.184
Singla et al. 2011	0.882	0.695	1.121	-1.025	0.305
De wit et al. 2011	0.887	0.704	1.118	-1.016	0.310
Cossar et al. 2008	0.905	0.743	1.102	-0.991	0.322
Alagarasu et al. 2007	0.877	0.696	1.104	-1.116	0.264
Soborg et al. 2007	0.883	0.709	1.100	-1.108	0.268
Liu et al. 2006	0.841	0.676	1.046	-1.555	0.120
Soborg et al. 2003	0.881	0.704	1.103	-1.102	0.270
Ozba? et al. 2003	0.871	0.698	1.088	-1.216	0.224
Selvaraj et al. 1999	0.844	0.678	1.050	-1.525	0.127
Hoal et al. 1999	0.927	0.775	1.110	-0.822	0.411
Bellamy et al. 1998	0.861	0.693	1.071	-1.345	0.179
	0.872	0.706	1.077	-1.272	0.203

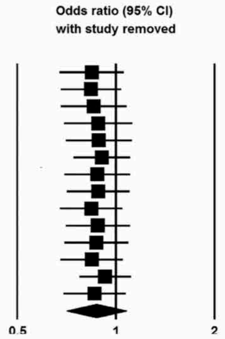

BB vs. AA+AB — Statistics with study removed

Study name	Point	Lower limit	Upper limit	Z-Value	p-Value
Wu et al. 2015	0.852	0.671	1.081	-1.321	0.186
Heidi et al. 2013	0.847	0.674	1.064	-1.427	0.153
Araujo et al. 2013	0.862	0.677	1.097	-1.208	0.227
Singla et al. 2011	0.898	0.704	1.147	-0.859	0.390
De wit et al. 2011	0.889	0.696	1.134	-0.949	0.343
Cossar et al. 2008	0.914	0.742	1.128	-0.836	0.403
Alagarasu et al. 2007	0.873	0.684	1.114	-1.089	0.276
Soborg et al. 2007	0.893	0.710	1.124	-0.963	0.335
Liu et al. 2006	0.845	0.672	1.063	-1.435	0.151
Soborg et al. 2003	0.880	0.693	1.116	-1.055	0.291
Ozba? et al. 2003	0.885	0.702	1.117	-1.029	0.304
Selvaraj et al. 1999	0.838	0.674	1.042	-1.591	0.112
Hoal et al. 1999	0.938	0.770	1.142	-0.639	0.523
Bellamy et al. 1998	0.866	0.690	1.089	-1.230	0.219
	0.878	0.703	1.095	-1.153	0.249

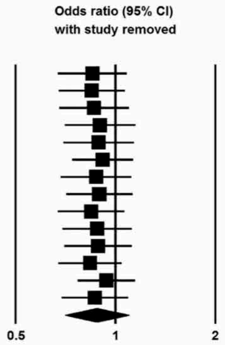

BB+AB vs. AA — Statistics with study removed

Study name	Point	Lower limit	Upper limit	Z-Value	p-Value
Wu et al. 2015	1.152	0.738	1.800	0.623	0.533
Heidi et al. 2013	1.144	0.736	1.779	0.599	0.549
Singla et al. 2011	0.748	0.427	1.308	-1.020	0.308
De wit et al. 2011	1.196	0.776	1.844	0.810	0.418
Cossar et al. 2008	1.162	0.754	1.791	0.682	0.495
Alagarasu et al. 2007	1.314	0.826	2.091	1.154	0.249
Soborg et al. 2007	1.145	0.743	1.764	0.612	0.541
Liu et al. 2006	1.236	0.788	1.939	0.923	0.356
Soborg et al. 2003	1.258	0.806	1.962	1.011	0.312
Ozba? et al. 2003	1.131	0.733	1.745	0.558	0.577
Selvaraj et al. 1999	1.292	0.833	2.003	1.146	0.252
Hoal et al. 1999	1.151	0.746	1.777	0.636	0.525
	1.166	0.759	1.789	0.700	0.484

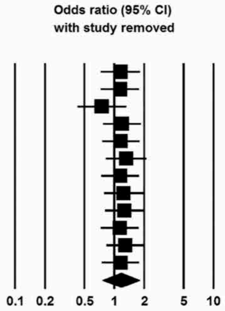

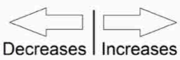

Figure SI1. Sensitivity analysis of MBL2 rs1800450 (A>B) gene polymorphism and TB risk to evaluate the influence of each individual study on the pooled OR by deleting one single study each time for overall analysis (for all the genetic models). Black square represents the value of OR and the size of the square indicates the inverse proportion relative to its variance. Horizontal line is the 95% CI of OR.

Study name<br>C vs. A	Point	Lower limit	Upper limit	Z-Value	p-Value
		**Statistics with study removed**			
Heidi et al. 2013	0.928	0.862	0.999	-1.996	0.046
Araujo et al. 2013	0.934	0.868	1.006	-1.809	0.070
Thye et al. 2011	0.945	0.837	1.066	-0.922	0.357
De wit et al. 2011	0.939	0.871	1.011	-1.667	0.096
Alagarasu et al. 2007	0.939	0.873	1.012	-1.655	0.098
Soborg et al. 2007	0.933	0.864	1.008	-1.764	0.078
Fitness et al. 2004	0.924	0.855	0.998	-2.018	0.044
Soborg et al. 2003	0.937	0.870	1.008	-1.738	0.082
Selvaraj et al. 1999	0.930	0.864	1.001	-1.936	0.053
Hoal et al. 1999	0.934	0.868	1.006	-1.809	0.070
Bellamy et al. 1998	0.957	0.885	1.035	-1.096	0.273
	0.936	0.869	1.007	-1.775	0.076

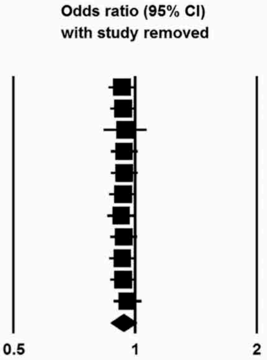

Study name<br>CC vs. AA	Point	Lower limit	Upper limit	Z-Value	p-Value
		**Statistics with study removed**			
Thye et al. 2011	0.782	0.555	1.101	-1.411	0.158
Alagarasu et al. 2007	0.837	0.701	1.000	-1.965	0.049
Soborg et al. 2007	0.826	0.687	0.994	-2.026	0.043
Fitness et al. 2004	0.831	0.692	0.998	-1.979	0.048
Soborg et al. 2003	0.829	0.694	0.990	-2.068	0.039
Selvaraj et al. 1999	0.836	0.700	0.998	-1.982	0.048
Bellamy et al. 1998	0.865	0.716	1.045	-1.503	0.133
	0.834	0.699	0.996	-2.002	0.045

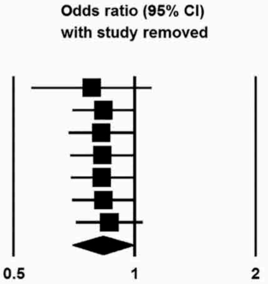

Study name<br>CA vs. AA	Point	Lower limit	Upper limit	Z-Value	p-Value
		**Statistics with study removed**			
Heidi et al. 2013	0.948	0.814	1.104	-0.688	0.492
Araujo et al. 2013	0.992	0.818	1.203	-0.079	0.937
Thye et al. 2011	1.041	0.802	1.350	0.299	0.765
De wit et al. 2011	1.027	0.832	1.269	0.251	0.802
Alagarasu et al. 2007	1.022	0.839	1.246	0.219	0.827
Soborg et al. 2007	1.018	0.811	1.276	0.151	0.880
Fitness et al. 2004	0.978	0.788	1.213	-0.204	0.838
Soborg et al. 2003	1.013	0.840	1.220	0.132	0.895
Selvaraj et al. 1999	0.959	0.812	1.133	-0.492	0.623
Hoal et al. 1999	0.996	0.816	1.216	-0.038	0.970
Bellamy et al. 1998	1.054	0.854	1.301	0.487	0.626
	0.999	0.827	1.208	-0.007	0.994

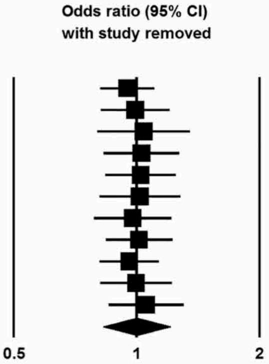

Study name<br>AA vs.CC+AC	Point	Lower limit	Upper limit	Z-Value	p-Value
		**Statistics with study removed**			
Heidi et al. 2013	0.930	0.811	1.067	-1.034	0.301
Araujo et al. 2013	0.972	0.811	1.166	-0.305	0.760
Thye et al. 2011	1.020	0.797	1.305	0.158	0.874
De wit et al. 2011	1.002	0.822	1.223	0.024	0.981
Alagarasu et al. 2007	1.000	0.831	1.204	0.002	0.998
Soborg et al. 2007	0.994	0.802	1.232	-0.055	0.956
Fitness et al. 2004	0.959	0.782	1.176	-0.401	0.688
Soborg et al. 2003	0.991	0.826	1.189	-0.096	0.923
Selvaraj et al. 1999	0.942	0.803	1.104	-0.743	0.457
Hoal et al. 1999	0.975	0.808	1.177	-0.263	0.793
Bellamy et al. 1998	1.032	0.850	1.253	0.315	0.752
	0.978	0.818	1.170	-0.239	0.811

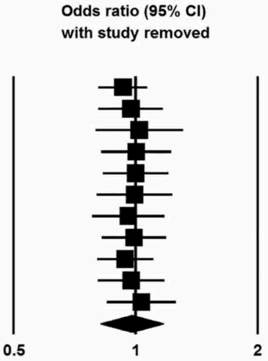

Study name<br>AA+AC vs. CC	Point	Lower limit	Upper limit	Z-Value	p-Value
		**Statistics with study removed**			
Thye et al. 2011	1.223	0.875	1.709	1.176	0.240
Alagarasu et al. 2007	1.156	0.975	1.371	1.669	0.095
Soborg et al. 2007	1.168	0.979	1.393	1.722	0.085
Fitness et al. 2004	1.157	0.971	1.379	1.636	0.102
Soborg et al. 2003	1.166	0.983	1.382	1.768	0.077
Selvaraj et al. 1999	1.157	0.976	1.372	1.679	0.093
Bellamy et al. 1998	1.133	0.945	1.357	1.348	0.178
	1.159	0.978	1.374	1.703	0.089

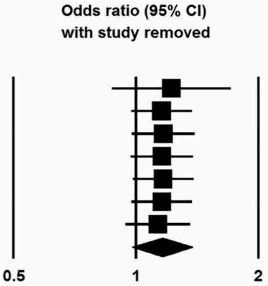

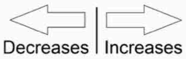

Figure SI2. Sensitivity analysis of MBL2 rs1800451 (A>C) gene polymorphism and TB risk to evaluate the influence of each individual study on the pooled OR by deleting one single study each time for overall analysis (for all the genetic models). Black square represents the value of OR and the size of the square indicates the inverse proportion relative to its variance. Horizontal line is the 95% CI of OR.

D vs. A — Statistics with study removed; Odds ratio (95% CI) with study removed

Study name	Point	Lower limit	Upper limit	Z-Value	p-Value
Heidi et al. 2013	1.127	0.812	1.565	0.716	0.474
Araujo et al. 2013	1.127	0.821	1.548	0.741	0.459
De wit et al. 2011	1.263	0.906	1.761	1.377	0.168
Alagarasu et al. 2007	1.039	0.747	1.446	0.229	0.819
Soborg et al. 2007	1.132	0.824	1.554	0.765	0.444
Soborg et al. 2003	1.141	0.811	1.604	0.757	0.449
Selvaraj et al. 1999	1.120	0.804	1.561	0.671	0.502
Hoal et al. 1999	1.185	0.872	1.611	1.087	0.277
	1.141	0.842	1.546	0.849	0.396

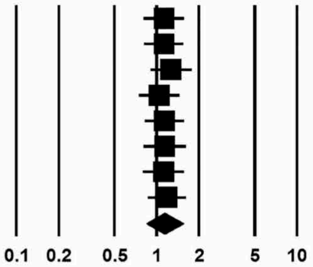

DD vs. AA — Statistics with study removed; Odds ratio (95% CI) with study removed

Study name	Point	Lower limit	Upper limit	Z-Value	p-Value
Heidi et al. 2013	3.676	0.699	19.323	1.537	0.124
Alagarasu et al. 2007	2.520	0.497	12.784	1.116	0.265
Soborg et al. 2003	2.742	0.522	14.404	1.192	0.233
Selvaraj et al. 1999	1.794	0.348	9.262	0.698	0.485
	2.592	0.623	10.780	1.310	0.190

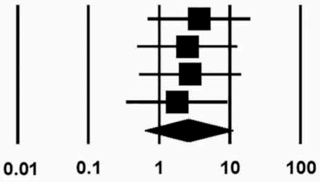

AD vs. AA — Statistics with study removed; Odds ratio (95% CI) with study removed

Study name	Point	Lower limit	Upper limit	Z-Value	p-Value
Heidi et al. 2013	0.928	0.653	1.319	-0.414	0.679
Araujo et al. 2013	0.942	0.668	1.327	-0.343	0.732
De wit et al. 2011	1.055	0.735	1.516	0.291	0.771
Alagarasu et al. 2007	0.873	0.611	1.249	-0.742	0.458
Soborg et al. 2007	0.946	0.671	1.333	-0.318	0.750
Soborg et al. 2003	0.950	0.660	1.368	-0.276	0.783
Selvaraj et al. 1999	1.072	0.756	1.520	0.389	0.698
Hoal et al. 1999	1.012	0.727	1.408	0.068	0.946
	0.971	0.700	1.346	0.178	0.859

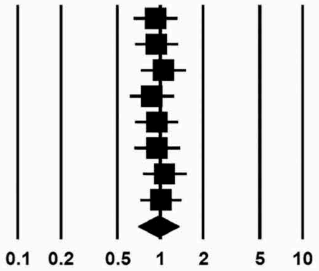

AA vs. DD+AD — Statistics with study removed; Odds ratio (95% CI) with study removed

Study name	Point	Lower limit	Upper limit	Z-Value	p-Value
Heidi et al. 2013	1.028	0.730	1.448	0.161	0.872
Araujo et al. 2013	1.037	0.744	1.445	0.212	0.832
De wit et al. 2011	1.165	0.821	1.652	0.854	0.393
Alagarasu et al. 2007	0.957	0.677	1.352	-0.250	0.803
Soborg et al. 2007	1.041	0.746	1.452	0.237	0.813
Soborg et al. 2003	1.047	0.735	1.491	0.253	0.800
Selvaraj et al. 1999	1.099	0.779	1.550	0.535	0.593
Hoal et al. 1999	1.102	0.799	1.519	0.592	0.554
	1.058	0.770	1.454	0.347	0.728

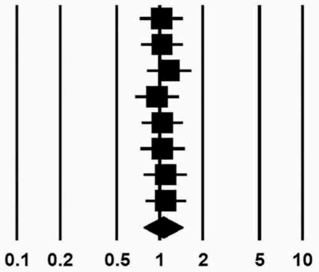

AA+AD vs.DD — Statistics with study removed; Odds ratio (95% CI) with study removed

Study name	Point	Lower limit	Upper limit	Z-Value	p-Value
Heidi et al. 2013	0.273	0.052	1.435	-1.533	0.125
Alagarasu et al. 2007	0.394	0.078	1.998	-1.124	0.261
Soborg et al. 2003	0.367	0.070	1.929	-1.184	0.237
Selvaraj et al. 1999	0.572	0.111	2.951	-0.667	0.505
	0.389	0.094	1.616	-1.300	0.194

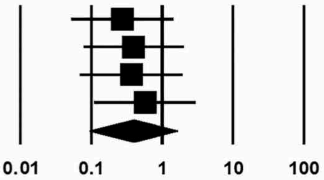

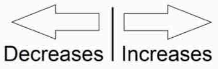

Figure SI3. Sensitivity analysis of rs5030737 (A>D) gene polymorphism and TB risk to evaluate the influence of each individual study on the pooled OR by deleting one single study each time for overall analysis (for all the genetic models). Black square represents the value of OR and the size of the square indicates the inverse proportion relative to its variance. Horizontal line is the 95% CI of OR.

Odds ratio (95% CI) with study removed

Study name O vs. A	Point	Lower limit	Upper limit	Z-Value	p-Value
Garcia et al. 2014	1.303	0.883	1.925	1.332	0.183
Heidi et al. 2013	1.273	0.857	1.891	1.194	0.232
Araujo et al. 2013	1.328	0.892	1.978	1.399	0.162
Capparelli et al. 2009	1.133	0.913	1.407	1.131	0.258
Alagarasu et al. 2007	1.324	0.881	1.989	1.351	0.177
Soborg et al. 2007	1.368	0.927	2.020	1.579	0.114
Garcia et al. 2006	1.422	0.993	2.036	1.923	0.055
Selvaraj et al. 2006	1.274	0.867	1.872	1.234	0.217
Soborg et al. 2003	1.332	0.896	1.981	1.417	0.156
Selvaraj et al. 1999	1.269	0.853	1.886	1.176	0.239
	1.303	0.909	1.869	1.441	0.150

Statistics with study removed

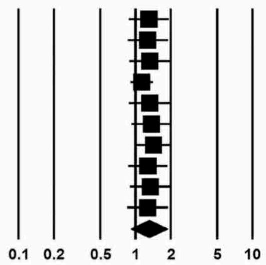

Odds ratio (95% CI) with study removed

Study name OO vs. AA	Point	Lower limit	Upper limit	Z-Value	p-Value
Garcia et al. 2014	2.086	0.817	5.329	1.537	0.124
Heidi et al. 2013	2.348	0.868	6.352	1.681	0.093
Araujo et al. 2013	2.334	0.898	6.066	1.739	0.082
Capparelli et al. 2009	1.581	0.852	2.932	1.452	0.147
Alagarasu et al. 2007	2.308	0.824	6.460	1.592	0.111
Soborg et al. 2007	2.638	1.057	6.588	2.078	0.038
Garcia et al. 2006	2.824	1.145	6.963	2.255	0.024
Selvaraj et al. 2006	2.218	0.843	5.834	1.614	0.106
Soborg et al. 2003	2.220	0.816	6.035	1.562	0.118
Selvaraj et al. 1999	1.998	0.774	5.168	1.430	0.153
	2.246	0.920	5.483	1.776	0.076

Statistics with study removed

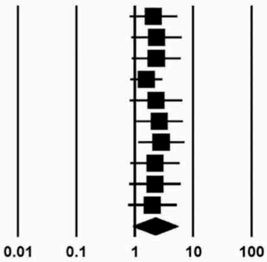

Odds ratio (95% CI) with study removed

Study name AO vs. AA	Point	Lower limit	Upper limit	Z-Value	p-Value
Garcia et al. 2014	1.244	0.830	1.865	1.056	0.291
Heidi et al. 2013	1.166	0.779	1.745	0.749	0.454
Araujo et al. 2013	1.240	0.816	1.884	1.008	0.313
Capparelli et al. 2009	1.025	0.838	1.253	0.239	0.811
Alagarasu et al. 2007	1.266	0.834	1.922	1.106	0.269
Soborg et al. 2007	1.265	0.821	1.949	1.066	0.287
Garcia et al. 2006	1.317	0.893	1.944	1.388	0.165
Selvaraj et al. 2006	1.196	0.804	1.779	0.881	0.378
Soborg et al. 2003	1.289	0.862	1.928	1.236	0.216
Selvaraj et al. 1999	1.228	0.811	1.861	0.971	0.332
	1.224	0.842	1.779	1.061	0.289

Statistics with study removed

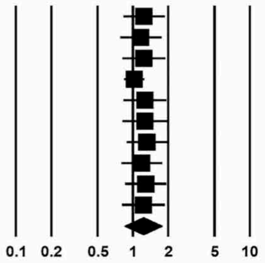

Odds ratio (95% CI) with study removed

Study name OO vs. AA+AO	Point	Lower limit	Upper limit	Z-Value	p-Value
Garcia et al. 2014	1.336	0.847	2.108	1.246	0.213
Heidi et al. 2013	1.273	0.804	2.015	1.029	0.303
Araujo et al. 2013	1.348	0.844	2.153	1.249	0.212
Capparelli et al. 2009	1.086	0.868	1.358	0.723	0.470
Alagarasu et al. 2007	1.360	0.847	2.184	1.271	0.204
Soborg et al. 2007	1.385	0.860	2.231	1.340	0.180
Garcia et al. 2006	1.449	0.941	2.229	1.685	0.092
Selvaraj et al. 2006	1.287	0.823	2.014	1.105	0.269
Soborg et al. 2003	1.379	0.870	2.185	1.367	0.172
Selvaraj et al. 1999	1.305	0.819	2.080	1.121	0.262
	1.321	0.868	2.011	1.298	0.194

Statistics with study removed

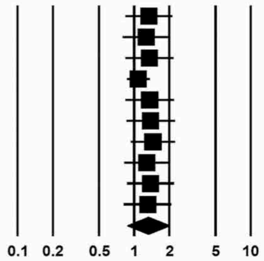

Odds ratio (95% CI) with study removed

Study name OO+AO vs. AA	Point	Lower limit	Upper limit	Z-Value	p-Value
Garcia et al. 2014	0.541	0.256	1.140	-1.615	0.106
Heidi et al. 2013	0.478	0.215	1.059	-1.818	0.069
Araujo et al. 2013	0.490	0.228	1.054	-1.824	0.068
Capparelli et al. 2009	0.648	0.359	1.167	-1.447	0.148
Alagarasu et al. 2007	0.499	0.218	1.141	-1.647	0.100
Soborg et al. 2007	0.431	0.213	0.871	-2.346	0.019
Garcia et al. 2006	0.416	0.204	0.852	-2.398	0.016
Selvaraj et al. 2006	0.507	0.233	1.102	-1.715	0.086
Soborg et al. 2003	0.523	0.236	1.162	-1.591	0.112
Selvaraj et al. 1999	0.567	0.268	1.199	-1.486	0.137
	0.504	0.246	1.033	-1.871	0.061

Statistics with study removed

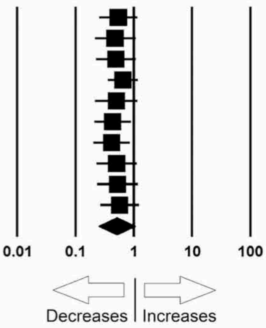

Figure SI4. Sensitivity analysis of MBL2 combined rs1800450, rs1800451, rs5030737 (A>O) exon 1 gene polymorphisms and TB risk to evaluate the influence of each individual study on the pooled OR by deleting one single study each time for overall analysis (for all the genetic models). Black square represents the value of OR and the size of the square indicates the inverse proportion relative to its variance. Horizontal line is the 95% CI of OR.

X vs. Y

Study name	Point	Lower limit	Upper limit	Z-Value	p-Value
Wu et al. 2015	1.104	0.884	1.378	0.873	0.383
Chen et al. 2015	1.108	0.878	1.398	0.862	0.388
Chen et al. 2014	1.072	0.877	1.311	0.682	0.495
Heidi et al. 2013	1.100	0.888	1.363	0.875	0.382
Thye et al. 2011	1.174	0.919	1.500	1.281	0.200
Alagarasu et al. 2007	1.206	1.016	1.432	2.139	0.032
Liu et al. 2006	1.104	0.888	1.372	0.890	0.373
	1.124	0.922	1.370	1.157	0.247

(Statistics with study removed)

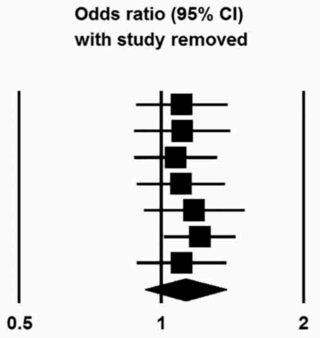

XX vs. YY

Study name	Point	Lower limit	Upper limit	Z-Value	p-Value
Wu et al. 2015	0.838	0.598	1.175	-1.023	0.306
Chen et al. 2015	0.872	0.620	1.227	-0.785	0.432
Chen et al. 2014	0.908	0.653	1.263	-0.574	0.566
Heidi et al. 2013	0.899	0.648	1.249	-0.634	0.526
Thye et al. 2011	0.862	0.579	1.282	-0.735	0.463
Alagarasu et al. 2007	1.069	0.757	1.510	0.380	0.704
Liu et al. 2006	0.865	0.621	1.203	-0.862	0.388
	0.900	0.655	1.235	-0.653	0.514

(Statistics with study removed)

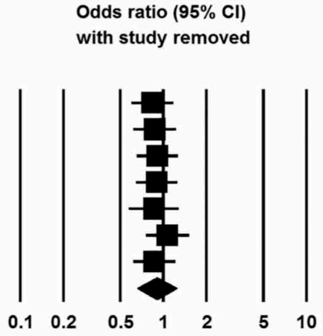

XY vs. XX

Study name	Point	Lower limit	Upper limit	Z-Value	p-Value
Wu et al. 2015	1.232	0.928	1.637	1.444	0.149
Chen et al. 2015	1.223	0.911	1.641	1.338	0.181
Chen et al. 2014	1.135	0.908	1.420	1.111	0.267
Heidi et al. 2013	1.190	0.917	1.544	1.305	0.192
Thye et al. 2011	1.321	1.005	1.736	1.994	0.046
Alagarasu et al. 2007	1.342	1.037	1.736	2.235	0.025
Liu et al. 2006	1.223	0.928	1.612	1.431	0.152
	1.235	0.964	1.582	1.671	0.095

(Statistics with study removed)

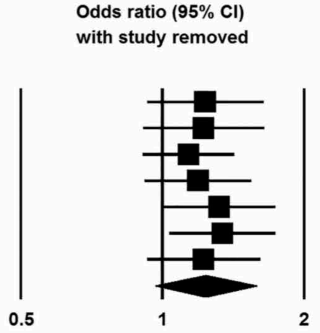

YY vs. XX+XY

Study name	Point	Lower limit	Upper limit	Z-Value	p-Value
Wu et al. 2015	1.185	0.900	1.559	1.211	0.226
Chen et al. 2015	1.182	0.888	1.573	1.145	0.252
Chen et al. 2014	1.116	0.886	1.405	0.931	0.352
Heidi et al. 2013	1.161	0.897	1.503	1.131	0.258
Thye et al. 2011	1.271	0.960	1.684	1.673	0.094
Alagarasu et al. 2007	1.308	1.035	1.654	2.243	0.025
Liu et al. 2006	1.180	0.903	1.542	1.211	0.226
	1.198	0.941	1.526	1.469	0.142

(Statistics with study removed)

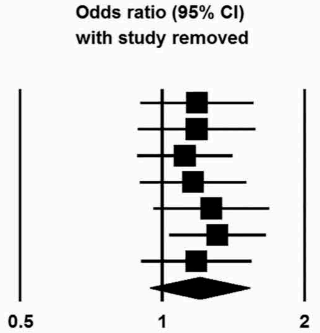

YY+XY vs. XX

Study name	Point	Lower limit	Upper limit	Z-Value	p-Value
Wu et al. 2015	1.226	0.877	1.713	1.191	0.234
Chen et al. 2015	1.174	0.837	1.646	0.928	0.354
Chen et al. 2014	1.121	0.808	1.555	0.682	0.495
Heidi et al. 2013	1.140	0.823	1.579	0.789	0.430
Thye et al. 2011	1.229	0.830	1.820	1.030	0.303
Alagarasu et al. 2007	0.992	0.703	1.398	-0.048	0.962
Liu et al. 2006	1.189	0.857	1.650	1.036	0.300
	1.148	0.839	1.573	0.862	0.389

(Statistics with study removed)

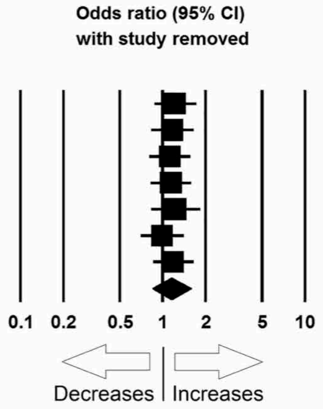

Figure SI5. Sensitivity analysis of MBL2 rs7096206 (Y>X) gene polymorphism and TB risk to evaluate the influence of each individual study on the pooled OR by deleting one single study each time for overall analysis (for all the genetic models). Black square represent the value of OR and the size of the square indicates the inverse proportion relative to its variance. Horizontal line is the 95% CI of OR.

Study name	Statistics with study removed				
H vs. L	Point	Lower limit	Upper limit	Z-Value	p-Value
Wu et al. 2015	1.047	0.914	1.198	0.659	0.510
Heidi et al. 2013	0.979	0.861	1.113	-0.328	0.743
Thye et al. 2011	0.956	0.806	1.136	-0.508	0.611
Liu et al. 2006	1.076	0.944	1.227	1.096	0.273
	1.019	0.904	1.149	0.309	0.757

Odds ratio (95% CI) with study removed

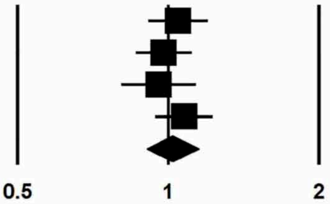

Study name	Statistics with study removed				
HH vs. LL	Point	Lower limit	Upper limit	Z-Value	p-Value
Wu et al. 2015	0.940	0.608	1.452	-0.281	0.779
Heidi et al. 2013	0.763	0.534	1.092	-1.477	0.140
Thye et al. 2011	0.904	0.638	1.281	-0.566	0.571
Liu et al. 2006	1.090	0.735	1.617	0.430	0.667
	0.907	0.653	1.260	-0.581	0.561

Odds ratio (95% CI) with study removed

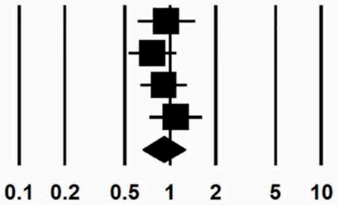

Study name	Statistics with study removed				
LH vs. LL	Point	Lower limit	Upper limit	Z-Value	p-Value
Wu et al. 2015	1.057	0.699	1.598	0.261	0.794
Heidi et al. 2013	0.789	0.499	1.247	-1.016	0.310
Thye et al. 2011	0.834	0.457	1.522	-0.590	0.555
Liu et al. 2006	0.992	0.567	1.735	-0.027	0.979
	0.905	0.588	1.394	-0.45[illegible]	0.[illegible]51

Odds ratio (95% CI) with study removed

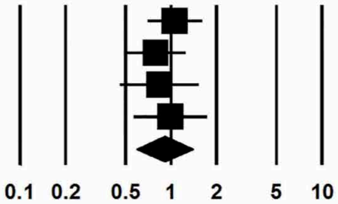

Study name	Statistics with study removed				
HH vs. LL+LH	Point	Lower limit	Upper limit	Z-Value	p-Value
Wu et al. 2015	1.065	0.661	1.717	0.259	0.795
Heidi et al. 2013	0.815	0.549	1.212	-1.010	0.312
Thye et al. 2011	0.887	0.492	1.600	-0.398	0.690
Liu et al. 2006	1.049	0.646	1.704	0.194	0.846
	0.935	0.626	1.396	-0.329	0.742

Odds ratio (95% CI) with study removed

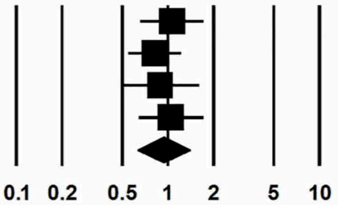

Study name	Statistics with study removed				
HH+LH vs. LL	Point	Lower limit	Upper limit	Z-Value	p-Value
Wu et al. 2015	0.988	0.719	1.357	-0.074	0.941
Heidi et al. 2013	0.998	0.735	1.355	-0.012	0.990
Thye et al. 2011	0.904	0.695	1.174	-0.757	0.449
Liu et al. 2006	0.803	0.598	1.077	-1.466	0.143
	0.915	0.710	1.179	-0.689	0.491

Odds ratio (95% CI) with study removed

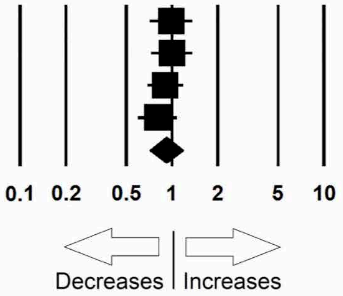

Figure SI6. Sensitivity analysis of MBL2 rs11003125 (H>L) gene polymorphism and TB risk to evaluate the influence of each individual study on the pooled OR by deleting one single study each time for overall analysis (for all the genetic models). Black square represents the value of OR and the size of the square indicates the inverse proportion relative to its variance. Horizontal line is the 95% CI of OR.

Study name — **Statistics with study removed** — **Odds ratio (95% CI) with study removed**

P vs. Q

Study name	Point	Lower limit	Upper limit	Z-Value	p-Value
Wu et al. 2015	1.022	0.937	1.115	0.498	0.619
Thye et al. 2011	0.876	0.624	1.230	-0.766	0.444
Liu et al. 2006	1.024	0.939	1.116	0.539	0.590
	1.018	0.935	1.109	0.414	0.679

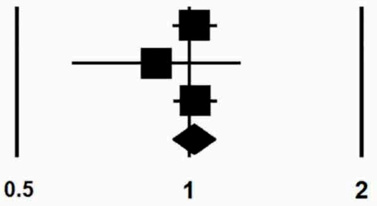

Study name — **Statistics with study removed** — **Odds ratio (95% CI) with study removed**

PP vs. QQ

Study name	Point	Lower limit	Upper limit	Z-Value	p-Value
Wu et al. 2015	1.096	0.911	1.319	0.971	0.332
Thye et al. 2011	1.032	0.188	5.673	0.036	0.971
Liu et al. 2006	1.101	0.915	1.324	1.015	0.310
	1.098	0.913	1.320	0.992	0.321

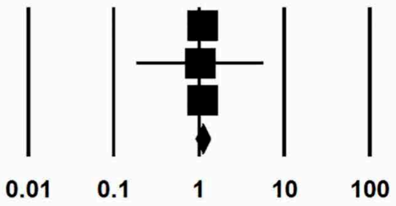

Study name — **Statistics with study removed** — **Odds ratio (95% CI) with study removed**

PQ vs. PP

Study name	Point	Lower limit	Upper limit	Z-Value	p-Value
Wu et al. 2015	0.956	0.840	1.088	-0.683	0.495
Thye et al. 2011	0.852	0.589	1.232	-0.851	0.395
Liu et al. 2006	0.958	0.842	1.089	-0.662	0.508
	0.950	0.839	1.077	-0.797	0.426

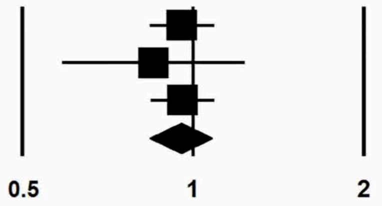

Study name — **Statistics with study removed** — **Odds ratio (95% CI) with study removed**

QQ vs. PP+PQ

Study name	Point	Lower limit	Upper limit	Z-Value	p-Value
Wu et al. 2015	0.985	0.871	1.114	-0.240	0.810
Thye et al. 2011	0.857	0.596	1.233	-0.831	0.406
Liu et al. 2006	0.987	0.874	1.115	-0.204	0.839
	0.979	0.869	1.102	-0.352	0.725

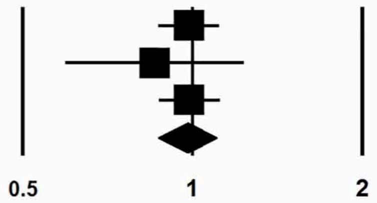

Study name — **Statistics with study removed** — **Odds ratio (95% CI) with study removed**

QQ+PQ vs. PP

Study name	Point	Lower limit	Upper limit	Z-Value	p-Value
Wu et al. 2015	0.893	0.754	1.058	-1.305	0.192
Thye et al. 2011	0.941	0.171	5.170	-0.069	0.945
Liu et al. 2006	0.890	0.751	1.055	-1.344	0.179
	0.892	0.753	1.056	-1.325	0.185

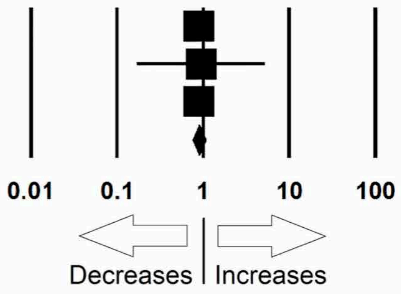

Figure SI7. Sensitivity analysis of rs7095891 (P>Q) gene polymorphism and TB risk to evaluate the influence of each individual study on the pooled OR by deleting one single study each time for overall analysis (for all the genetic models). Black square represents the value of OR and the size of the square indicates the inverse proportion relative to its variance. Horizontal line is the 95% CI of OR.

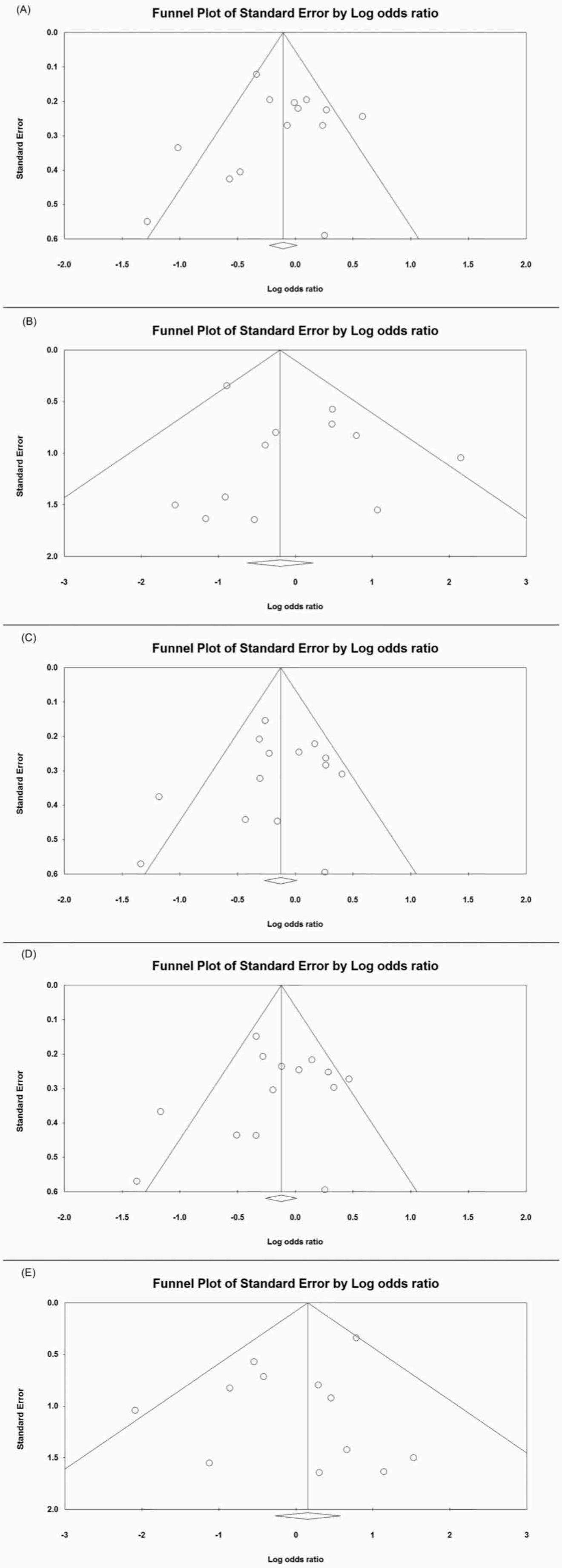

Figure SI8. Assessment of publication bias shown with Funnel plots in studies assaying odds of TB associated with the MBL2 rs1800450 (A>B) gene polymorphism for overall analysis (odds ratio against standard error in different genetic models); (A) B vs. A; (B) BB vs. AA; (C) AB vs. AA; (D) BB vs. AA+AB; (E) BB+AB vs. AA

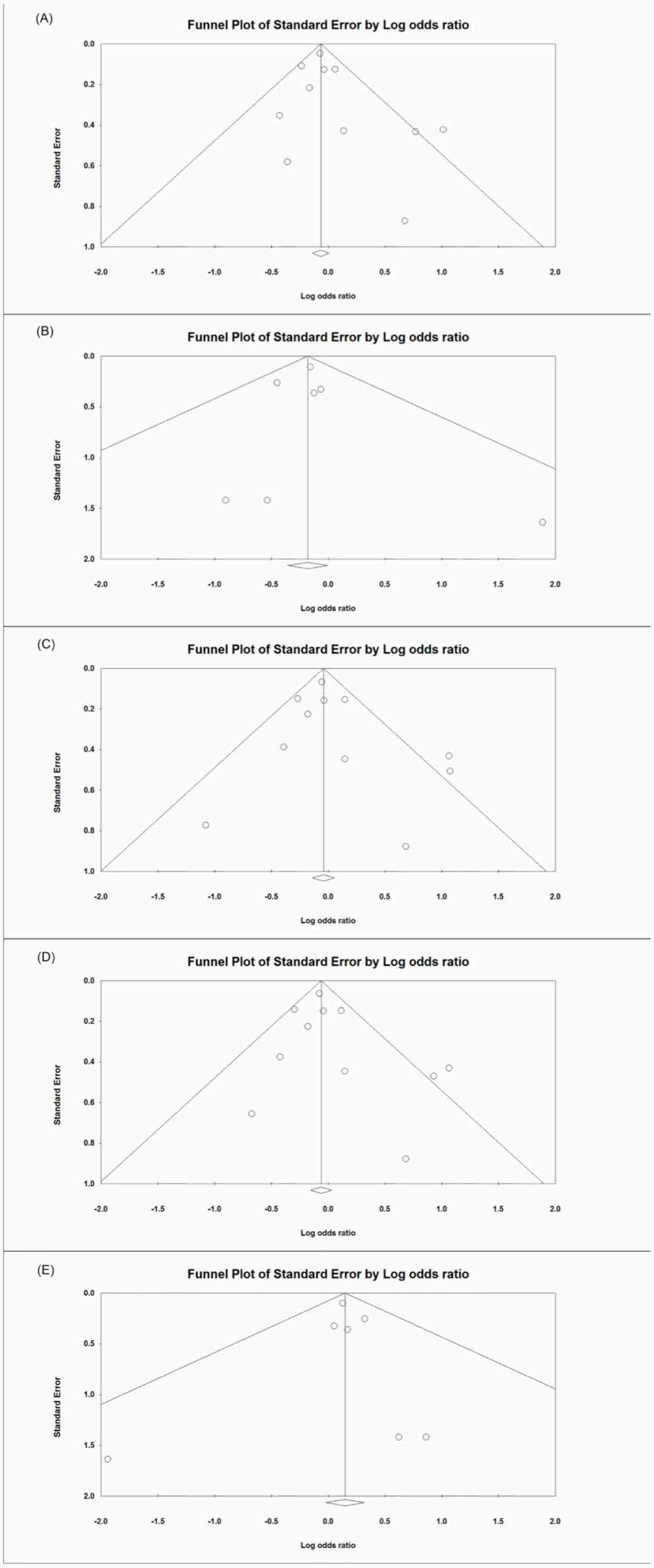

Figure SI9. Assessment of publication bias shown with Funnel plots in studies assaying odds of TB associated with the MBL2 rs1800451 (A>C) gene polymorphism for overall analysis (Odds ratio against standard error in different genetic models. (A) C vs. A; (B) CC vs. AA; (C) CA vs. AA; (D) AA vs. CC+AC; (E) AA+AC vs. CC.

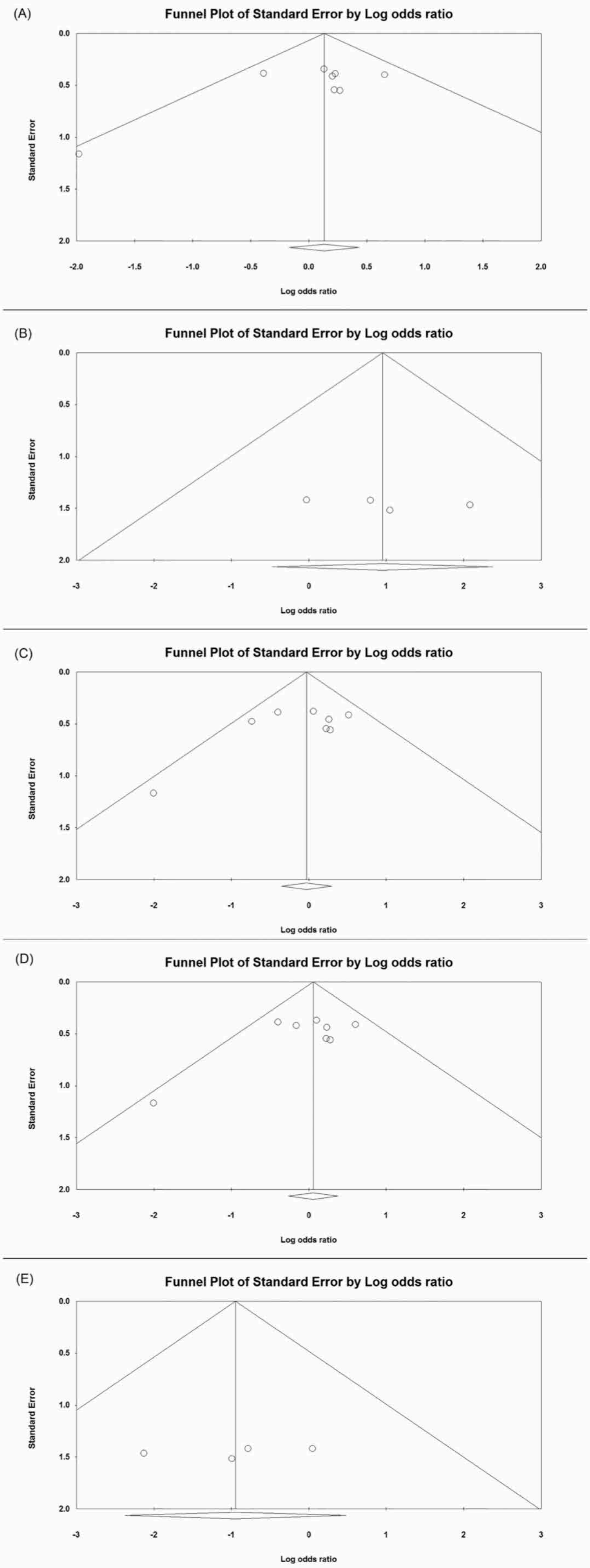

Figure SI10. Assessment of publication bias shown with Funnel plots in studies assaying odds of TB associated with the MBL2 rs5030737 (A>D) gene polymorphism for overall analysis (Odds ratio against standard error in different genetic models. (A) D vs. A; (B) DD vs. AA; (C) AD vs. AA; (D) AA vs. DD+AD; (E) AA+AD vs. DD.

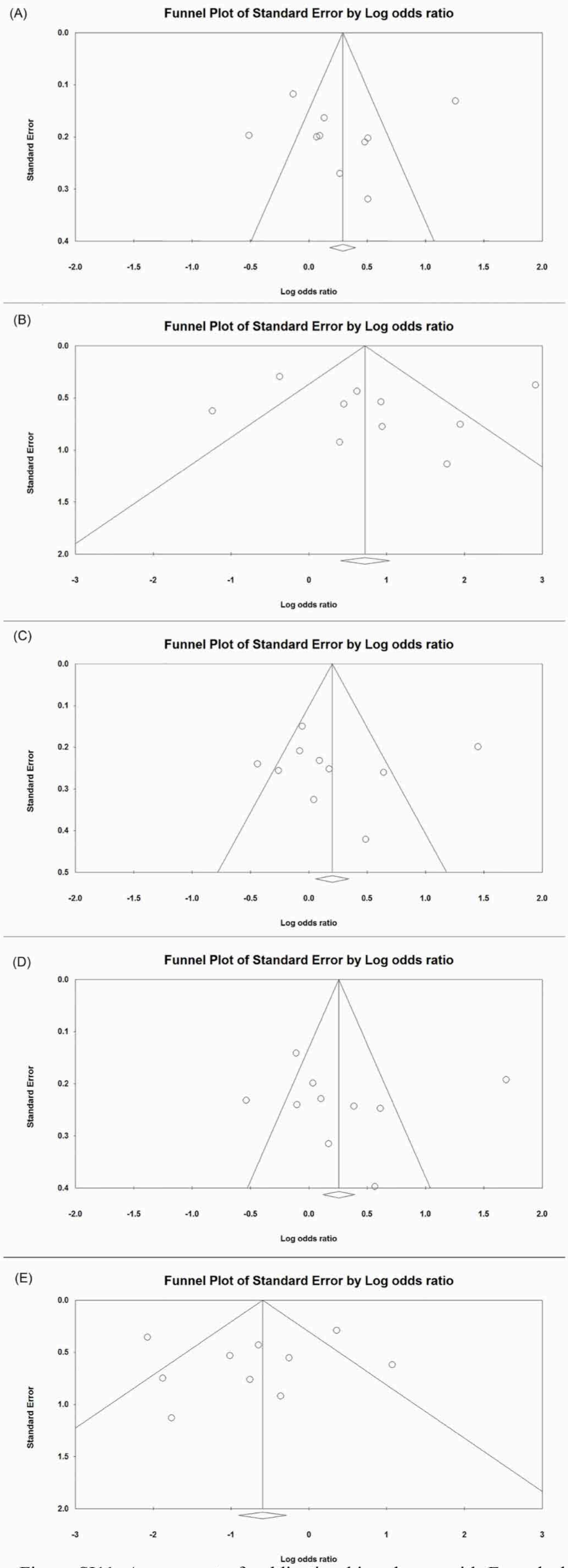

Figure SI11. Assessment of publication bias shown with Funnel plots in studies assaying odds of TB associated with the MBL2 combined rs1800450, rs1800451, 5030737 (A>O) exon 1 gene polymorphisms for overall analysis (Odds ratio against standard error in different genetic models. (A) O vs. A; (B) OO vs. AA; (C) AO vs. AA; (D) OO vs. AA+AO; (E) OO+AO vs. AA.

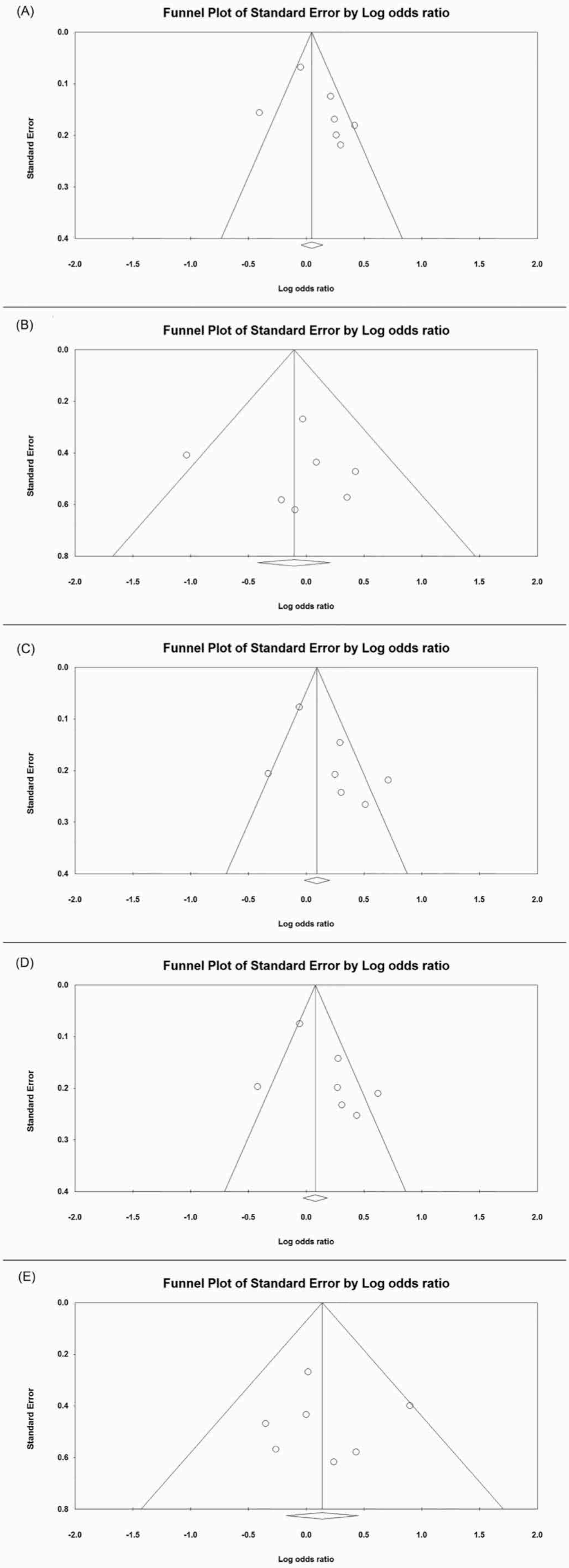

Figure SI12. Assessment of publication bias shown with Funnel plots in studies assaying odds of TB associated with the MBL2 rs7096206 (Y>X) gene polymorphism for overall analysis (Odds ratio against standard error in different genetic models. (A) X vs. Y; (B) XX vs. YY; (C) XY vs. XX; (D) YY vs. XX+XY; (E) YY+XY vs. XX.

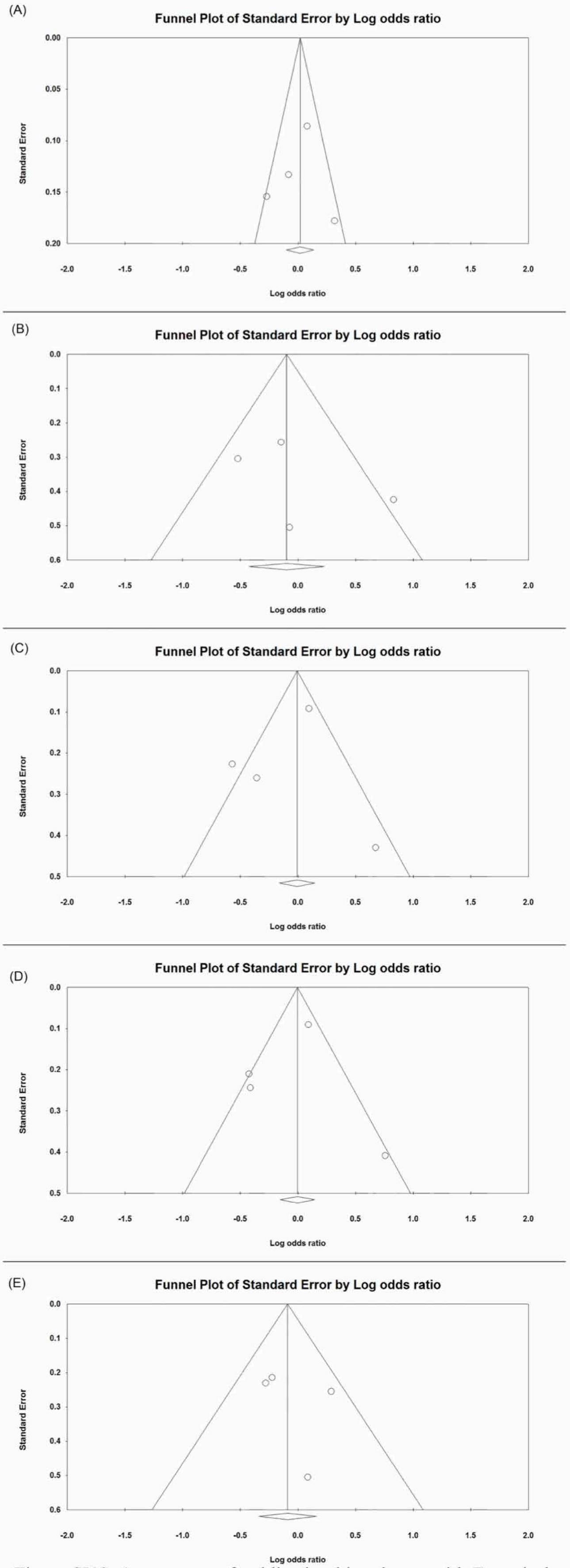

Figure SI13. Assessment of publication bias shown with Funnel plots in studies assaying odds of TB associated with the MBL2 rs11003125 (H>L) gene polymorphism for overall analysis (Odds ratio against standard error in different genetic models. (A) H vs. L; (B) HH vs. LL; (C) LH vs. LL; (D) HH vs. LL+LH; (E) HH+LH vs. LL.

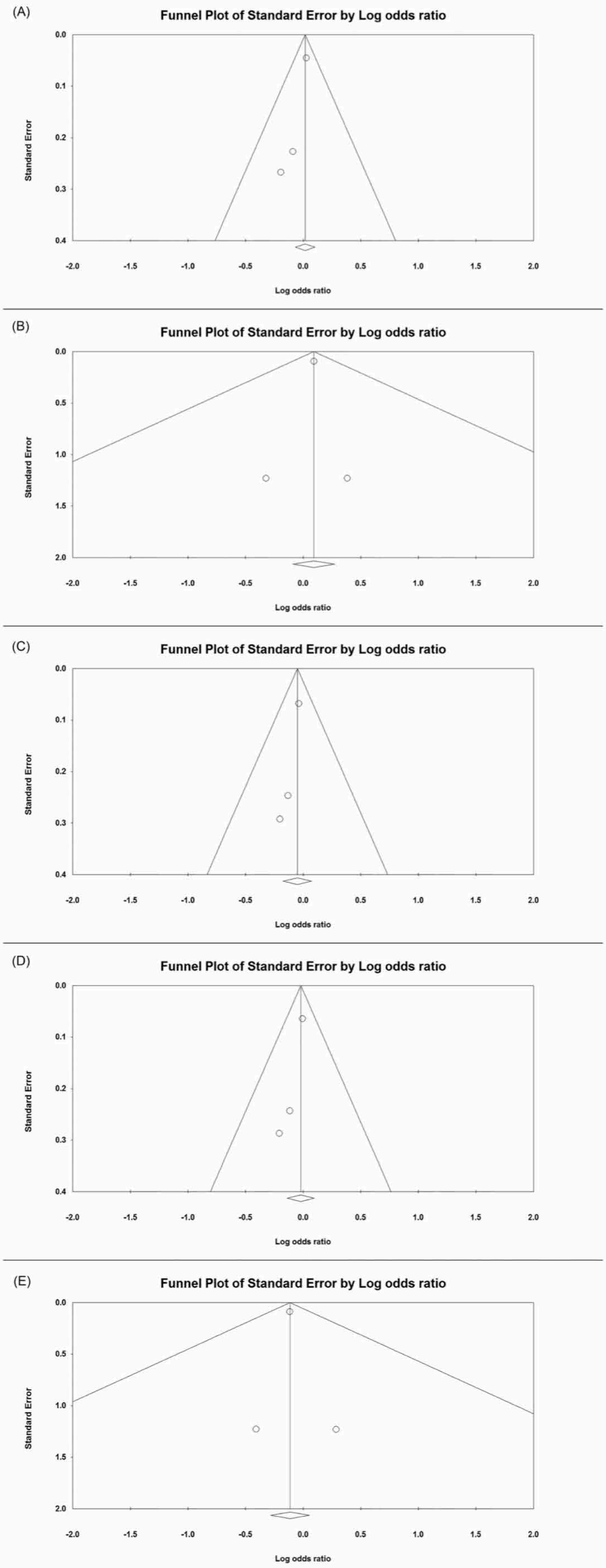

Figure SI14. Assessment of publication bias shown with Funnel plots in studies assaying odds of TB associated with the MBL2 rs7095891 (P>Q) gene polymorphism for overall analysis (Odds ratio against standard error in different genetic models. (A) Q vs. P; (B) QQ vs. PP; (C) PQ vs. PP; (D) QQ vs. PP+PQ; (E) QQ+PQ vs. PP.

Table ST1. Statistics to test publication bias and heterogeneity in the meta-analysis (rs1800450 African population)

Comparisons	Egger's regression analysis			Heterogeneity analysis			Model used for the meta-analysis
	Intercept	95% Confidence Interval	p-value	Q-value	$P_{heterogeneity}$	I^2 (%)	
B vs. A	-0.40	-9.57 to 8.76	0.86	5.59	0.13	46.36	Fixed
BB vs. AA	0.98	-141.43 to 143.40	0.94	1.23	0.53	0.001	Fixed
AB vs. AA	-0.13	-9.59 to 9.32	0.95	5.64	0.13	46.86	Fixed
BB vs. AA+AB	0.10	-136.71 to 136.92	0.99	1.16	0.55	0.001	Fixed
BB+AB vs. AA	-0.30	-9.98to9.38	0.90	5.90	0.11	49.15	Fixed

Table ST2. Statistics to test publication bias and heterogeneity in the meta-analysis (rs1800450 Asian population)

Comparisons	Egger's regression analysis			Heterogeneity analysis			Model used for the meta-analysis
	Intercept	95% Confidence Interval	p-value	Q-value	$P_{heterogeneity}$	I^2 (%)	
B vs. A	6.19	2.74 to9.64	0.01	14.8	0.005	73.05	Random
BB vs. AA	3.48	-0.08to7.05	0.05	11.33	0.023	64.69	Random
AB vs. AA	3.61	-2.03to9.26	0.13	5.96	0.20	32.96	Fixed
BB vs. AA+AB	-3.16	-6.80 to 0.46	0.06	10.20	0.03	60.79	Random
BB+AB vs. AA	5.59	0.92 to 10.26	0.03	9.94	0.04	59.79	Random

Table ST3. Statistics to test publication bias and heterogeneity in the meta-analysis (rs1800451 African population)

Comparisons	Egger's regression analysis			Heterogeneity analysis			Model used for the meta-analysis
	Intercept	95% Confidence Interval	p-value	Q-value	$P_{heterogeneity}$	I^2 (%)	
C vs. A	0.10	-1.88 to 2.09	0.88	3.88	0.56	0.01	Fixed
CC vs. AA	-0.24	-3.57 to 3.07	0.77	1.24	0.74	0.01	Fixed
CA vs. AA	0.07	-2.27 to 2.42	0.93	4.31	0.50	0.01	Fixed
CC+CA vs. AA	0.11	-2.27 to 2.50	0.90	4.66	0.45	0.01	Fixed
CC vs. AA+CA	0.24	-1.94 to 2.42	0.67	0.59	0.89	0.01	Fixed

Table ST4. Statistics to test publication bias and heterogeneity in the meta-analysis (rs7096206 Asian population)

Comparisons	Egger's regression analysis			Heterogeneity analysis			Model used for the meta-analysis
	Intercept	95% Confidence Interval	p-value	Q-value	$P_{heterogeneity}$	I^2 (%)	
X vs. Y	1.80	-17.50 to 21.11	0.78	15.62	0.004	74.40	Random
XX vs. YY	4.35	-9.31 to 18.01	0.38	7.24	0.12	44.81	Fixed
XY vs. XX	0.12	-15.67 to 15.93	0.98	12.40	0.01	67.75	Random
XY+XX vs. YY	0.03	-17.78 to 17.84	0.99	14.57	0.006	72.55	Random
XX vs. XY+YY	3.21	-8.73 to 15.16	0.45	5.47	0.24	26.98	Fixed
